# Global variation in soil carbon sequestration potential through improved cropland management

**DOI:** 10.1111/gcb.15954

**Published:** 2021-11-12

**Authors:** Malte Lessmann, Gerard H. Ros, Madaline D. Young, Wim de Vries

**Affiliations:** ^1^ Land Use Planning Group Wageningen University and Research Wageningen The Netherlands; ^2^ Environmental Systems Analysis Group Wageningen University and Research Wageningen the Netherlands

**Keywords:** climate change mitigation, global carbon sequestration potential, management impacts, meta‐analysis, soil organic carbon

## Abstract

Widespread adoption of improved cropland management measures is advocated to increase soil organic carbon (SOC) levels, thereby improving soil fertility and mitigating climate change. However, spatially explicit insight on management impacts is limited, which is crucial for region‐specific and climate‐smart practices. To overcome these limitations, we combined global meta‐analytical results on improved management practices on SOC sequestration with spatially explicit data on current management practices and potential areas for the adoption of these measures. We included (a) fertilization practices, i.e., use of organic fertilizer compared to inorganic fertilizer or no fertilizer, (b) soil tillage practices, i.e., no‐tillage relative to high or intermediate intensity tillage, and (c) crop management practices, i.e., use of cover crops and enhanced crop residue incorporation. We show that the estimated global C sequestration potential varies between 0.44 and 0.68 Gt C yr^−1^, assuming maximum complementarity among all measures taken. A more realistic estimate, not assuming maximum complementarity, is from 0.28 to 0.43 Gt C yr^−1^, being on the lower end of the current range of 0.1–2 Gt C yr^−1^ found in the literature. One reason for the lower estimate is the limited availability of manure that has not yet been recycled. Another reason is the limited area for the adoption of improved measures, considering their current application and application limitations. We found large regional differences in carbon sequestration potential due to differences in yield gaps, SOC levels, and current practices applied. The highest potential is found in regions with low crop production, low initial SOC levels, and in regions where livestock manure and crop residues are only partially recycled. Supporting previous findings, we highlight that to encourage both soil fertility and SOC sequestration, it is best to focus on agricultural soils with large yield gaps and/or where SOC values are below levels that may limit crop production.

## Graphical abstract

Widespread adoption of improved cropland management is needed to increase soil organic carbon (SOC) levels. We compiled information from global meta‐analysis studies reporting SOC changes due to improved cropland management. We combined this data with spatial information defining the areas where these measures have the potential to be adopted. Our findings suggest that the global SOC sequestration potential from improved management ranges between 0.28 and 0.43 Gt C yr^‐1^, being on the lower end of the current range of 0.1–2 Gt C yr^‐1^ found in literature.

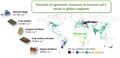

## 1 INTRODUCTION

### 1.1 Impacts of agricultural management on soil carbon sequestration

Agricultural soils are under considerable threat due to unsustainable cultivation practices. Intensive fertilization, tillage, and monocultures have led to negative impacts on soil quality (Chemnitz & Weigelt, 2015), including a loss in soil organic carbon (SOC). On a global scale, it is estimated that the SOC stock of arable soils is depleted by 25–75% compared to the antecedent SOC stock (Lal, 2013). SOC has important functions such as water retention or nutrient supply (Vogel et al., 2018) and enhances soil biological and physical properties such as aggregate stability and disease suppressiveness (Lal, 2013; Schlatter et al., 2017). Within a broad range of soil properties, SOC has been widely used as an indicator to evaluate soil quality in response to management impacts under various environmental conditions (Bünemann et al., 2018).

Practices such as green manuring, increased cereal use in rotation schemes, inorganic fertilizer use or substitution inorganic with organic fertilizers, crop residue incorporation, and reduced tillage have been increasingly promoted to elevate SOC levels (Bolinder et al., 2020; Singh et al., 2018; Spiegel et al., 2014). The importance of SOC sequestration in arable soils on climate change mitigation and food security has also been recognized at the COP 21 in France in 2015, where the “4 per mille Soils for Food Security and Climate” initiative was launched by the French Ministry of Agriculture. The original rationale was to counteract the annual rise in atmospheric CO_2_, estimated at 4.3 Gt C yr^−1^ in the period 2003–2012 (Le Quéré et al., 2014). The desired reduction originated from an estimated emission decline of 0.9 Gt C yr^−1^ from halting deforestation and an annual 0.4% increase in SOC in the top 40 cm of all non‐permafrost, agricultural, and non‐agricultural soils (http://4p1000.org/). Since then, however, the global potential of SOC sequestration has been questioned, as the potential is likely limited to unfertilized, non‐agricultural land (Van Groenigen et al., 2017; de Vries, 2018).

### 1.2 Global soil carbon sequestration potential of agricultural management practices

Current estimates of the global SOC sequestration potential of agricultural management in cropland range between 0.1 and 2 Gt C yr^−1^. Fuss et al. (2018) provided a summary of 22 articles with global SOC potentials from improved management based on per‐area sequestration estimates. Their synthesis suggested that individual practices applied to global croplands have a potential ranging from 0.4 to 0.8 Gt C yr^−1^. These estimates were based on a variety of global studies making assumptions on the areas available and the type of practices applied. For instance, Zomer et al. (2017) suggested global estimates of 0.9–1.85 Gt C yr^−1^, assuming that practices including cover cropping, manure application, and reduced tillage could sequester about 0.56–1.15 t C ha^–1^ yr^–1^. Paustian et al. (2016) suggested lower ranges of about 0.08–0.4 Gt C yr^−1^ for improved cropland management including reduced tillage, improved nutrient management, and crop rotation. For the adoption of no tillage, Powlson et al. (2014) estimated an annual accumulation rate of 0.3 t C ha^–1^ yr^–1^ given increases of 0.17 Gt C yr^−1^ when applied to a global cereal cropping area of 559 Mha (excluding current no till areas). For cover crop cultivation, Poeplau and Don (2015) provided an estimate of 0.12 Gt C yr^−1^ assuming that cover crops are used in 25% of the total cropland area (400 Mha).

More recently, Minasny et al. (2017) evaluated sequestration potentials for a range of management impacts in 20 world regions and found a global sequestration potential of 2–3 Gt C yr^−1^ in the top 1 m of the soil profile. However, their estimates were criticized by de Vries (2018) due to the inclusion of around 5000 Mha of non‐managed arable land and grassland, where there is a lack of nutrients such as N and P to sequester the carbon. In view of stoichiometric considerations for C, N, and P, their estimate was considered implausible (Van Groenigen et al., 2017; de Vries, 2018). Similarly, SOC potentials by Paustian et al. (2016) and Smith et al. (2008) were likely overestimated due to their assumptions made on the area available for improved management practices.

Apart from stoichiometric considerations, there are various other limitations in the earlier estimates on global C sequestration potentials from improved management. First, estimates are often limited to individual practices, such as no tillage (Powlson et al., 2014) or cover crop cultivation (Poeplau & Don, 2015), or no distinction is made between specific management measures (Zomer et al., 2017). Second, studies providing estimates for management impacts on SOC do often not account for the appropriate areas available to apply these practices. For example, in the case of organic manure application and crop residue incorporation, potentials are limited by the amount of manure or crop residues that is not yet recycled or incorporated. Moreover, for assessing the global impact of reduced or no tillage, better insight is needed into the current application of (conventional) tillage and the potentially suitable areas where it may be reduced (Porwollik et al., 2019). Similar reasoning holds for crop diversification and crop residue use.

Considering the above‐mentioned limitations, Amelung et al. (2020) stressed that the 4p1000 initiative is an aspirational goal due to its dependency on land use, soil, and climatic regions, but also inspirational since opportunities for climate change mitigation may go hand in hand with improvements in soil health and food production security. They proposed the establishment of a soil information system to assess SOC sequestration potentials, with a focus on regions where nutrient management would enhance both crop production and SOC.

### 1.3 Aim of this study

This paper focuses on the potential to sequester carbon in soil via improved cropland management (estimated at 1510–1611 Mha or 11–12% of total global land; Lambin and Meyfroidt (2011)). Insight on the expected spatial variation of SOC response to management measures in croplands at a global scale would be highly desirable in view of the global‐scale soil climate mitigation strategy advocated by Amelung et al. (2020). Until now, such an insight is lacking. This study aims to extend our insights by combining results of recent global meta‐studies on SOC changes due to improved agricultural measures with spatial information on their potential application in croplands. More specifically, we did so by: (a) collecting reported meta‐analytical effect sizes on SOC changes in the soil in response to improved fertilization, reduced tillage, increased crop rotation, and improved crop residue management in different climate zones and (b) multiplying this with an estimate of the area where each measure can be applied, considering their current application and application limitations. By doing so, we come up with reliable ranges in SOC sequestration for each practice as a function of regional site properties and more realistic estimates of the potential global SOC sequestration in croplands.

## 2 MATERIALS AND METHODS

### 2.1 Review of meta‐analytical studies

#### 2.1.1 Data collection

Effects of agricultural management impacts on SOC and related site‐specific factors were retrieved from published meta‐studies and systematic reviews, focusing on cropland on non‐peaty soils. Peer‐reviewed publications were collected using the advanced search function in Scopus to identify matches for the title, abstract and keywords of papers, with search strings organized by: study type, subject, interventions, and measured the response given in Table S1. Further synthesis data were included from the European Catch‐C project report (Spiegel et al., 2014).

After bibliometric querying, publications were assessed based on a pre‐defined set of criteria. Studies were selected when including SOC changes from agronomic management for at least one of the defined management interventions listed in Table 1. We excluded reviews that were not based on results from long‐term field experiments. Moreover, studies that focused on combining effects of multiple management practices such as integrated farming were not retained. For the included studies, information was extracted on regional coverage (temperate, subtropical, tropical), site‐specific factors (soil properties, climatic conditions, management‐related factors), study duration (average, range), sampling depth (average, range), and corresponding changes on SOC. SOC stock changes (in t C ha^−1^, t C ha^−1^ yr^−1^) and concentration changes (in g C kg^−1^) were commonly reported as the raw mean difference between treatment and control group over a given study period. The relative change (% yr^−1^, %) was also recorded from the reported (response) ratio between treatment and control. Measures of variation were extracted as standard error, 95% confident interval, and standard deviation.

**TABLE 1:** Classification of management measures (M1–M5) and corresponding interventions in relation to original treatment and control groups from meta‐studies

Measure	Description measure	Intervention	Description intervention (treatment – control)	Original treatment description	Original control description
M1	Increased inorganic fertilization	IF‐NF	Inorganic fertilization – No fertilization	Balanced chemical N,P,K fertilizer; Unbalanced chemical N,P,K fertilizer; Synthetic N addition; N fertilizer addition; Mineral N fertilizer	No fertilizer
M2	Increased organic inputs	OF‐NF	Organic fertilization – No fertilization	Organic amendments; Manure application	No fertilizer
		OF‐IF	Organic fertilization – Inorganic fertilization	Organic amendments; Manure application; Bovine slurry application; Farmyard manure application	Conventional management; Mineral N fertilizer
		COF‐NF	Combined organic +inorganic fertilization – No fertilization	Manure +chemical N,P,K fertilizer; Organic +synthetic N fertilizer	No fertilizer
		COF‐IF	Combined organic +inorganic fertilization – Inorganic fertilization	Bovine slurry +mineral N fertilizer; Farmyard manure +mineral N fertilizer	Mineral N fertilizer
		CRF‐NF	Combined straw return +inorganic fertilization – No fertilization	Straw return +chemical N,P,K fertilizer	No fertilizer
M3	Reduced tillage	IT‐HT	Intermediate intensity till – High intensity till	Reduced till; Intermediate intensity till; Minimum and reduced non‐inversion till	Conventional till; Deep inversion till; High intensity till
		NT‐HT	No till – High intensity till	No till	Conventional till; Full inversion till; Ploughing; Inversion till
		NT‐IT	No till – Intermediate intensity till	No till	Intermediate intensity till
M4	Increased crop diversity	C	Increased rotations (excl. cover crops) – Monoculture	Crop rotations without cover crops	Monoculture
		CC	Increased rotations +cover crops – Monoculture	Cover cropped rotations; Enhancement of rotation complexity	Bare fallows; Rotations without cover crops; Monoculture (grain); No cover crops; Monoculture or bare fallows
		CCP	Perennial cropped rotations – Monoculture	Perennial cropped rotations	Monoculture (grain)
M5	Crop residue incorporation	CRES	Crop residue incorporation – Residue removal	Return of crop residues; Corn stover retention	Removal of crop residues

#### 2.1.2 Data retrieval

An overview of the original treatment and control groups from meta‐studies classified according to the management and intervention classes is provided in Table 1. Detailed information on the included meta‐studies for different measures, with original treatment and control group descriptions, is given in Table S2 and an overview of the identified studies classified per management and metric of SOC changes is provided in Table S3. A total of 20 meta‐studies and quantitative reviews were included analyzing agricultural management impacts on SOC for a wide range of management, climatic zones, soil types, and cropping systems on a global scale and Europe. While most studies focused on individual management impacts, a few studies addressed multiple interventions (Aguilera et al., 2013; Spiegel et al., 2014; West & Post, 2002). Additive effects on SOC due to combined management, however, were not assessed.

Increased inorganic fertilization (M1) included a wide variety of treatments such as (un) balanced chemical N, P, K fertilization, and N addition only (Table 1). Similarly, increased organic matter input (M2) included a wide range of treatments such as slurry, farmyard manure, and organic amendments. We added an additional intervention (straw return +chemical fertilizer vs. no fertilizer (CRF‐NF)) to the M2 measure based on the data provided by Han et al. (2016).

There was no uniform approach used by meta‐studies to assess changes in SOC (Table S3). Changes were expressed as percent (in % or % yr^−1^), concentration (in g kg^−1^ or g kg^−1^ yr^−1^), or stock (in t C ha^−1^ or t C ha^−1^ yr^−1^). SOC concentrations were usually provided when data on bulk densities were lacking (Han et al., 2016; Ladha et al., 2011; McDaniel et al., 2014). Measures of variation were most often reported as standard error or 95% confidence interval, and a few studies provided standard deviations. SOC changes per management and climate zone were extracted directly from tables and text if possible, or otherwise from figures using WebPlotDigitizer (Rohatgi, 2018). Other factors such as fertilizer application rates or soil types could not be accounted for due to lacking information. When no separate estimates on SOC changes per climate zone were provided, data were classified based on the geographic coverage of the study. Where possible, soil depth and study duration were recorded separately for each management intervention and climate zone, otherwise, the range or average was reported per management.

#### 2.1.3 Data analysis

To reach a uniform comparison and global upscaling approach, all data on SOC concentration changes were converted to annual stock changes in t C ha^−1^ yr^−1^. The Annual stock change was chosen in order to describe the absolute amount of carbon that has been lost or sequestered to and from the atmosphere. From the initial 20 meta‐studies, only 14 meta‐studies could be used to derive absolute values on stock changes as the remaining studies only provided estimates on relative SOC changes with missing data on initial stocks. Information on the studies that were ultimately used is given in Table S3. From those 14 studies, 11 studies provided original SOC stock change data, while it lacked in 3 studies, i.e., in Lu et al. (2011), partly in Haddaway et al. (2017) and Han et al. (2016). For Lu et al. (2011), we multiplied the average initial stock (t C ha^−1^) by the change in SOC (%) in response to inorganic N addition relative to no fertilization. The meta‐study by Haddaway et al. (2017) provided only part of the data on SOC stock changes, all data on SOC concentration changes as well as the field study coordinates. To include all their data, we converted the concentration changes into stock changes using spatially explicit and soil layer‐dependent bulk density estimates from the SoilGrids dataset (which provides global estimates at 250m resolution based on ca. 150,000 soil profiles; Hengl et al., 2014, 2017). More specifically, we transformed SOC concentrations to stocks by multiplying the soil depth (m) with the bulk density (kg m^−3^) and the given change in SOC concentration (g kg^−1^), giving the stock in g m^−2^, which is equal to 0.01 t C ha^−1^. The study by Han et al. (2016) lacks original field study coordinates; hence, we assigned an average bulk density value of 1400 kg m^−3^, considering a global range (>90% of the values within this range) of approximately 1200–1600 kg m^−3^ for mineral topsoils (0–30 cm) in SoilGrids (Hengl et al., 2017).

Secondly, meta‐study estimates were normalized by dividing the derived stock changes by an overall duration of 20 years to allow for a balanced evaluation and comparison of the impacts of measures. The reference period of 20 years was selected as it was the median duration from meta‐studies and can be considered a representative period to assess medium‐term impacts on SOC. This normalization came with the assumption that SOC accumulation is linear with time, which is a simplification since SOC accumulation has been identified as a first‐order process (van Groenigen et al., 2014). Poulton et al. (2018) showed that SOC saturation effects occurred after 80–100 years following farmyard manure application, while simulations by Poeplau and Don (2015) showed a new steady state for SOC stocks after 155 years following cover crop cultivation. Since saturation effects have not been reported by meta‐studies, we assume that a linear response on the mid‐term does not lead to substantial bias in our estimates. It is, however, likely that the normalization leads to underestimations in the short term (<5 years) and overestimations over the long term (>20 years). Third, all measures of variation were transformed into standard error (SE), using SE = SD/√n, when standard deviation (SD) and sample size n (number of paired comparisons) were provided by meta‐studies. When 95% confidence intervals (CI) were reported, the SE was calculated via CI/3.92. If studies did not report measures of variation, we included an arbitrary SD value based on a coefficient of variation (CV) of 1.25 times the average CV calculated per management‐climate combination. Finally, to assess the overall impact per management‐climate combination, we calculated the weighted group mean using inverse‐variance weighting (Tang et al., 2013). This entailed that when different meta‐studies covered the same management‐impact pair, we provided averages of the reported effect sizes, weighted by standard deviation following Eq. S1 and Eq. S2. Data on the absolute and relative SOC stock change thus derived from the various meta‐studies, with information on the conversions done from SOC concentration to SOC stock and from total SOC changes to annual changes, being given in Tables S7–S11.

### 2.2 Global upscaling of results

The meta‐analysis results were upscaled to global SOC sequestration potentials by multiplying the estimates per management and climate with the potential area where the practice could be applied (see Figure S1 for a schematic overview on the upscaling process). Here, we made a distinction between the most important driving factors, including fertilization level, cropping system, climate zone (tropical, subtropical, temperate), and currently applied management. For fertilization, we distinguished between non, medium, and highly fertilized systems where medium and highly fertilized croplands were further differentiated between systems with low and high animal manure inputs. The classification was based on different combinations of N fertilizer and N manure inputs (Table S4). For the other management measures, we classified cropland areas according to no, medium, and high‐intensity tillage systems, crop rotations (with and without cereals/catch crops), and crop residue treatments (with and without the potential for crop residue incorporation).

To estimate the areas where each of the measures could be applied, we combined spatially explicit data from the Koeppen Geiger classification map (Table S5) (Peel et al., 2007), the spatial production allocation model (SPAM) for land use (Wood‐Sichra et al., 2016), maps on N fertilizer application rates (Lu & Tian, 2016), N manure production and application rates on cropland and grassland (Xu, Tian, et al., 2019; Zhang et al., 2017), the global tillage system dataset (Porwollik et al., 2019) and FAO databases on cropping systems (FAO, 2020a), crop residue retention (FAO, 2020c), and crop residue burning (FAO, 2020b). Detailed information on the databases used and the integration and aggregation steps to derive the respective cropland areas is provided in the Supplementary methods. Following the cropland classification, we multiplied the meta‐analysis results with the respective areas given the differences in interventions, i.e., differences in treatment and control. For instance, areas identified with high‐intensity tillage (HT) and intermediate intensity tillage (IT) have the potential to be converted to no till (NT) given the estimated SOC impacts for the NT‐HT and NT‐IT comparisons (Table S6). Similarly, non‐fertilized areas (with N input <40 kg N ha^−1^) have the potential to increase SOC by inorganic fertilization given the IF‐NF impact. The increase in SOC due to organic fertilizers was maximized given the remaining animal manure that is not yet used in agriculture. To estimate this amount of recyclable animal C‐manure, we subtracted the N applied on cropland and grassland (including grazing) from the total N excreted (Xu, Tian, et al., 2019; Zhang et al., 2017), assuming a CN ratio of 10 (for the organic fraction) and an average humification coefficient of 0.5 for both slurry and solid manure (CBAV, 2020; Gobin et al., 2011; Veeken et al., 2017) with the humification coefficient being defined as the stable carbon fraction that is not decomposed within one year. The uncertainties in the global model estimates after upscaling were assessed via Monte Carlo analysis (n = 1000) using a normal distribution for the estimated SOC change due to a measure, where the mean response (as well as the variance) were derived from the weighted group mean SOC impact of a measure using inverse‐variance weighting (Tang et al., 2013; Young et al., 2020).

## 3 RESULTS

### 3.1 Carbon sequestration due to management measures: meta‐analysis results

#### 3.1.1 Overall impacts of management measures

**FIGURE 1:**
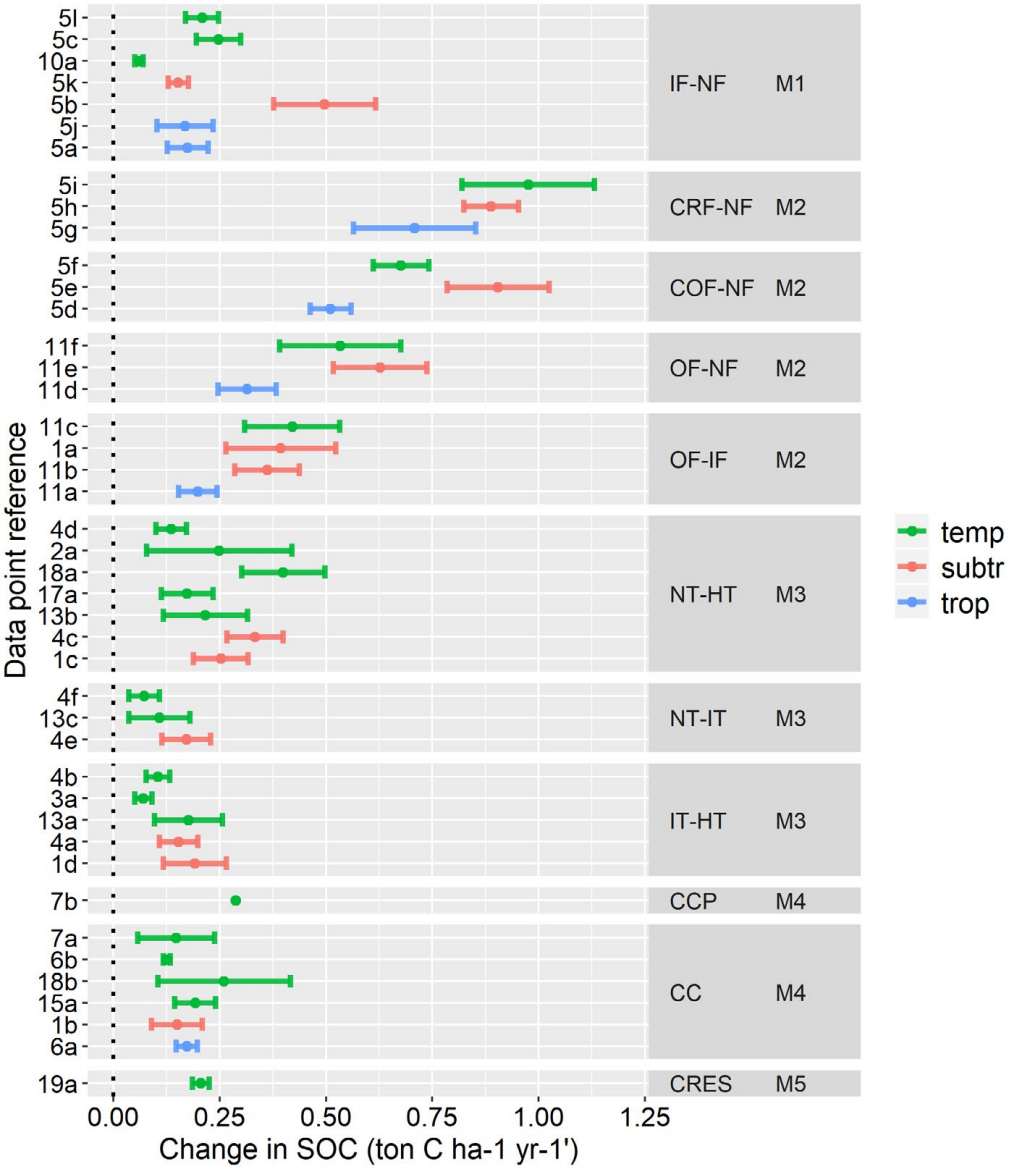
Impacts of improved agricultural management on soil carbon (SOC) stock changes (in t C ha‐1 yr‐1) in the top 20–30 cm soil depth continued over a timeframe of 20 years. Climate zones given in legend: temperate (temp), sub‐tropical (subtr) and tropical (trop). The reference numbers refer to the data derived from the meta‐studies listed in Table S2, where the letters indicate when multiple values came from the same study. Increased inorganic fertilization (M1): inorganic fertilizer ‐ no fertilizer (IF‐NF); Increased organic matter input (M2): Combined fertilizer relative to No Fertilizer (COF‐NF), Combined Straw +fertilizer relative to No Fertilizer (CRF‐NF), Organic Fertilizer relative to Inorganic Fertilizer (OF‐IF), Organic Fertilizer relative to No Fertilizer (OF‐NF); Decreased tillage (M3): No‐Till relative to High Intensity‐Till (NT‐HT), No‐Till relative to Intermediate Intensity‐Till (NT‐IT) and Intermediate Intensity‐Till relative to High Intensity‐Till (IT‐HT); Increased crop diversity (M4): Crop rotation +cover crops (CC) and perennial crop rotation (CCP); Crop residue incorporation (M5): crop residue incorporation vs. removal (CRES). The error bars represent the standard error. More detailed information on underlying data including sampling depths and study durations are provided in the Tables S7–S11

Current meta‐studies and quantitative reviews indicate a pronounced effect of improved fertilizer management on SOC stock compared to reduced tillage, crop rotation, and residue incorporation (Figure 1). The largest effects were found for combined straw and inorganic fertilizer application (CRF‐NF) with an overall change of 0.86 t C ha^−1^ yr^−1^, followed by combined organic and inorganic fertilization (COF‐NF) with 0.70 t C ha^−1^ yr^−1^, organic relative to no fertilization (OF‐NF) with 0.49 t C ha^−1^ yr^−1^, and organic relative to inorganic fertilization (OF‐IF) with 0.33 t C ha^−1^ yr^−1^. For reduced till, we found that no‐till relative to high intensity‐till (NT‐HT) increased SOC by 0.24 t C ha^−1^ yr^−1^, followed by intermediate intensity‐till relative to high intensity‐till (IT‐HT) with 0.14 t C ha^−1^ yr^−1^ and no‐till relative to intermediate intensity‐till (NT‐IT) with 0.13 t C ha^−1^ yr^−1^. With regard to increased crop diversity, we found that perennial cropped rotations (CCP) caused a larger overall change (0.29 t C ha^−1^ yr^−1^) than cover cropped rotations (CC) (0.15 t C ha^−1^ yr^−1^). Crop residue incorporation (CRES) showed an average SOC change of 0.21 t C ha^−1^ yr^−1^. For inorganic fertilization, we found that balanced fertilization strategies (with N,P,K) had the most pronounced effects of 0.33 t C ha^−1^ yr^−1^, followed by unbalanced strategies (one or two types of N,P,K) with 0.18 t C ha^−1^ yr^−1^ and inorganic N addition only with 0.06 t C ha^−1^ yr^−1^. Data on absolute SOC changes could not be derived for increased crop rotations vs. monoculture (C) and combined organic and inorganic relative to inorganic fertilizer application (COF‐IF). Detailed results of the synthesis data are provided in Tables S7–S11 for increased inorganic fertilization (M1), increased organic matter input (M2), reduced tillage (M3), increased crop diversity (M4), and crop residue incorporation (M5), respectively.

To assess the overall impact per management‐climate combination, we aggregated the SOC changes from Figure 1 by calculating the weighted group mean, giving more weight to estimates from larger meta‐studies (Table 2). The weighting did not affect SOC changes for the management‐climate groups, where multiple values were not given (i.e., CRF‐NF, COF‐NF, OF‐NF, CCP, CRES). For increased inorganic vs. no fertilization (IF‐NF), we estimated overall SOC changes of 0.17 t C ha^−1^ yr^−1^ for subtropical and tropical climates and 0.12 t C ha^−1^ yr^−1^ for temperate climates. For organic vs. inorganic fertilization (OF‐IF), the overall SOC changes were found to be 0.42 t C ha^−1^ yr^−1^ in temperate zones. For no till vs. high intensity till (NT‐HT), we derived weighted means of 0.28 and 0.19 t C ha^−1^ yr^−1^ for subtropical and temperate zones, respectively. For no till vs. intermediate till (NT‐IT), the weighted means were 0.17 and 0.09 t C ha^−1^ yr^−1^ in subtropical and temperate climates. Weighted means for intermediate intensity till vs. high intensity till (IT‐HT) were 0.17 and 0.10 t C ha^−1^ yr^−1^ in subtropical and temperate climates, respectively. For cover cropped rotations, we found an overall increase of 0.14 t C ha^−1^ yr^−1^ for temperate zones, where the values for tropical and subtropical zones stayed the same.

**TABLE 2:** Weighted mean SOC stock changes (t C ha^−1^ yr^−1^ ± standard error) per management and climate zone for the top 20‐30cm soil depth. Increased inorganic fertilization (M1): inorganic fertilizer—no fertilizer (IF‐NF); Increased organic matter input (M2): Combined fertilizer relative to No Fertilizer (COF‐NF), Combined Straw +fertilizer relative to No Fertilizer (CRF‐NF), Organic Fertilizer relative to Inorganic Fertilizer (OF‐IF), Organic Fertilizer relative to No Fertilizer (OF‐NF); Decreased tillage (M3): No‐Till relative to High Intensity‐Till (NT‐HT), No‐Till relative to Intermediate Intensity‐Till (NT‐IT) and Intermediate Intensity‐Till relative to High Intensity‐Till (IT‐HT); Increased crop diversity (M4): Crop rotation +cover crops (CC) and perennial crop rotation (CCP); Crop residue incorporation (M5): crop residue incorporation vs. removal (CRES)

Measure	Intervention Category	Weighted mean SOC stock changes in t C ha^−1^ yr^−1^ ± standard error			
		Temperate	Subtropical	Tropical	Other
M1	IF‐NF	0.12 ± 0.01	0.17 ± 0.02	0.17 ± 0.04	0.14 ± 0.01
M2	COF‐NF	0.68 ± 0.06	0.90 ± 0.12	0.51 ± 0.05	0.59 ± 0.04
	CRF‐NF	0.98 ± 0.16	0.89 ± 0.06	0.71 ± 0.14	0.85 ± 0.05
	OF‐IF	0.42 ± 0.11	0.37 ± 0.07	0.20 ± 0.04	0.29 ± 0.04
	OF‐NF	0.53 ± 0.14	0.63 ± 0.11	0.31 ± 0.07	0.43 ± 0.05
M3	IT‐HT	0.10 ± 0.02	0.17 ± 0.04	–	0.13 ± 0.01
	NT‐HT	0.19 ± 0.03	0.28 ± 0.05	–	0.23 ± 0.02
	NT‐IT	0.09 ± 0.03	0.17 ± 0.06	–	0.10 ± 0.03
M4	CC	0.14 ± 0.01	0.15 ± 0.06	0.17 ± 0.03	0.15 ± 0.01
	CCP	0.29 ± 0.19	–	–	0.29 ± 0.19
M5	CRES	0.21 ± 0.02	–	–	0.21 ± 0.02

We found the differences in SOC changes between management and climate zones (Figure 1, Table 2). Combined organic and inorganic fertilization (COF‐NF) and organic fertilization (OF‐NF) increased SOC levels along tropical<temperate<subtropical climate zones. For combined straw and inorganic fertilizer application (CRF‐NF) and organic vs. inorganic fertilization (OF‐IF), we observed an increase in SOC along tropical<subtropical<temperate. Increased inorganic fertilization showed the lowest SOC changes in temperate zones. Similarly, in the case of reduced tillage, the increase in SOC changes was lower in temperate compared to subtropical zones. No values for tropical climates could be derived. For increased crop diversity (CC), we found small differences in SOC along temperate<subtropical<tropical climates.

### 3.2 Global SOC sequestration potential

The total area of cropland used for upscaling was 1,416,912 thousand hectares. Approximately 30% of the cropland area was located in tropical climate zones, 19% in temperate zones, 30% in subtropical or Mediterranean zones, and the remaining 21% in other climatic zones. About 45% was characterized by high‐intensity tillage systems, whereas only 8% was cultivated with conservation agricultural practices minimizing soil tillage. In 25% of the cropland area, substantial amounts of crop residues were burned, whereas 72% of the crop rotations did not include catch crops. On most arable land, less than 40 kg N ha^−1^ came from animal manure, and between 40 and 100 kg N ha^−1^ came from inorganic fertilizers. Only 13% of all arable land received high levels of both organic and inorganic fertilizers.

The measure with the highest global potential to sequester carbon is the increased use of organic carbon inputs via organic fertilization, ignoring the total amount of animal manure that is available or already applied (Table 3), estimated to be an increase of 592 Mton C yr^−1^. The global manure nitrogen production from six livestock categories, including cattle, chickens, ducks, goats, swine, and sheep equaled 129 Tg N yr^−1^ in 2010, with data being available at 5‐arc minutes (Zhang et al., 2017). Assuming a C:N ratio of about 10, being the average C:N value for slurry (approximately 8) and solid manure (approximately 15), the total estimated C produced via animal manure is 1310 Mton C yr^−1^, but might range between 1048 and 1965 Mton C yr^−1^. However, only part of the manure excreted actually goes to cropland since the part is left on pasture due to grazing and part is burned as fuel. Since the currently applied manure does not lead to additional C sequestration in soil, we calculated the amount of manure available in each country currently being wasted or burned (see supplementary methods), being less than 10% of the total excreted amount. Considering the application of unused manure, this leads to a corrected C sequestration in soil use of up to 26–30 Mtons C yr^−1^.

**TABLE 3:** Potential C sequestration (in Mton C year^−1^) per measure and climate zone for the top 20–30 cm soil depth. Values between brackets are with 0.01 and 0.99 confidence intervals

Climate zone	M1	M2	M2cor	M3	M4	M5
	*Inorganic fertilizer*	*Organic fertilizer*	*Organic fertilizer*	*Tillage*	*Catch crops*	*Crop residue*
Subtropical‐Mediterranean	21 (18–23)	235 (142–326)	10 (6–10)	82 (50–114)	49 (2–96)	27 (21–33)
Temperate	9 (8–10)	114 (82–145)	6 (5–6)	42 (29–56)	33 (29–38)	22 (17–27)
Tropical	35 (27–45)	139 (85–199)	8 (8–8)	58 (39–77)	34 (23–45)	8 (6–10)
Other	13 (13–14)	104 (82–125)	6 (5–6)	45 (32–57)	37 (33–41)	16 (12–20)
all	78 (70–88)	592 (467–713)	30 (26–30)	227 (182–273)	153 (106–202)	73 (56–90)

The second important measure controlling the potential of soils to sequester C is the adoption of no‐till and minimum tillage practices in arable cropping systems. The total C sequestration ranges from 42 to 82 Mton yr^−1^ over the different climate zones, resulting in an estimated total C sequestration potential of 227 Mton yr^−1^. Increasing the inorganic N dose in arable systems increases the net C input by about 78 Mton C yr^−1^ via root C inputs in the rhizosphere (via root biomass and exudates) and via non‐harvestable crop residues leftover on the field after harvest. Increasing crop diversification as well as the use of catch crops and cereals within arable crop rotation schemes has the potential to sequester an additional 153 Mton of C yr^−1^. Since the incorporation of crop residues is a well‐established practice in most countries, the impact of this measure is limited to about 73 Mton C year^−1^.

**FIGURE 2:**
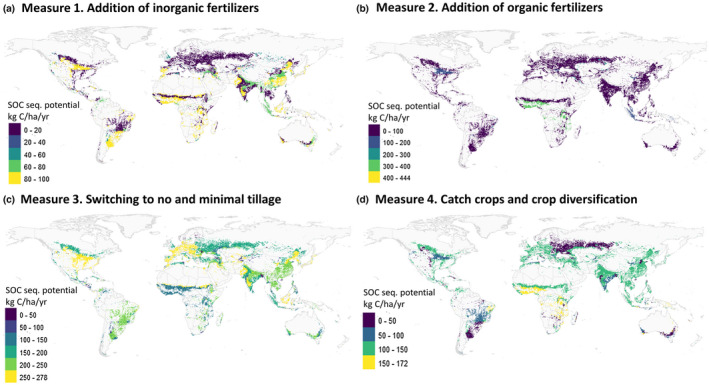
The soil organic carbon (SOC) sequestration potential (in kg C /ha arable land / year) estimated in the top 20–30 cm soil depth for four agronomic measures (a) addition of inorganic fertilizers, (b) addition of organic fertilizers, (c) switching to no and minimal tillage and (d) catch crops and crop diversification as estimated from meta‐analytical field experiments and extrapolated to all arable agro‐ecosystems given the climate zone, soil tillage practices, and crop rotation system. The legend differs per map

Assuming that the individual evaluated measures mentioned here are complementary to each other, the total C sequestration potential might increase up to 561 Mton C year^−1^ on arable systems worldwide. Using a simple weighting factor where the additive contribution declines with the order of the measure when sorted from highest to lowest impact, the total C sequestration potential is about 354 Mton C year^−1^ and could vary from 278 to 432 Mton C year^−1^. The highest impact of inorganic fertilization is visible in parts of Africa and Southern and North America due to the expected yield increase from additional nitrogen inputs. Minimal soil tillage has the biggest impact in agricultural regions that have the potential to shift away from intensive tillage systems, and these occur over the whole globe with high potential for the USA, Europe, and parts of Northern Africa (Figure 2). Crop diversification and the use of catch crops are applicable in most regions.

## 4 DISCUSSION

### 4.1 Impacts of management on SOC stock changes

#### 4.1.1 Overall impacts

In our study, we made use of reported meta‐data to approximate overall SOC changes in soils in response to a wide range of management measures and different climate zones. The overall impacts of improved cropland management on SOC ranged on an average from 0.2 to 0.6 t C ha^−1^ yr^−1^. Compared to other global figures, literature shows comparable ranges of 0.2–0.4 t C ha^−1^ yr^−1^ (Dawson & Smith, 2007) and 0.2–0.5 t C ha^−1^ yr^−1^ (Minasny et al., 2017), as well as higher values of 0.56–1.15 t C ha^–1^ yr^−1^ (Zomer et al., 2017). Organic matter inputs led to the highest mean SOC changes (0.6 t C ha^–1^ yr^−1^), followed by crop residue incorporation (0.21 t C ha^–1^ yr^−1^), reduced tillage (0.18 t C ha^–1^ yr^−1^), increased crop diversity (0.18 t C ha^–1^ yr^−1^), and increased inorganic fertilization (0.15 t C ha^–1^ yr^−1^). In comparison, the synthesis of reviews by Bolinder et al. (2020) found the highest average SOC changes from manure application (~0.4 t C ha^−1^ yr^−1^), followed by cover crops (~0.3 t C ha^−1^ yr^−1^), nitrogen fertilization (~0.23 t C ha^−1^ yr^−1^), and crop residue retention (~0.12 t C ha^−1^ yr^−1^). Minasny et al. (2017) found the highest SOC changes from organic amendments (~0.5 t C ha^−1^ yr^−1^), followed by residue incorporation (~0.35 C ha^−1^ yr^−1^), no or reduced till (~0.3 t C ha^−1^ yr^−1^), and crop rotation (~0.2 t C ha^–1^ yr^−1^).

When looking into the effects of the management interventions, we observed more pronounced effects from combined organic and inorganic vs. no fertilization (COF‐NF), compared to organic vs. no fertilization (OF‐NF) and organic vs. inorganic fertilization (OF‐IF) with 0.70, 0.49, and 0.33 t C ha^−1^ yr^−1^, respectively. The overall positive influence of inorganic fertilization on SOC with 0.2 t C ha^−1^ yr^−1^ could explain the difference in SOC between the management classes (COF‐NF vs. OF‐NF). The magnitude of this effect, however, strongly depends on the type of organic and inorganic fertilizers considered (Figure 1, Tables S7 and S8). Moreover, while the effects of increased organic matter inputs on SOC are directly linked to the type and amount of material applied, SOC responses to inorganic fertilizer application are indirectly linked to increased crop productivity resulting in higher carbon inputs (Geisseler & Scow, 2014; Poffenbarger et al., 2017). Furthermore, Han et al. (2016) indicated that inorganic fertilizers and straw mulching interacted, leading to increased SOC changes potentially due to the positive effects of straw application on enhanced nitrogen use efficiencies increasing crop productivity and therefore enhancing C inputs. Aguilera et al. (2013) did not find higher SOC changes for the combination of compost and cover cropping when compared to compost application only. Based on these findings, it is not clear whether the impacts of management are additive since the effects on SOC can be different per management and a variety of interactions might play a role. Though the impact of inorganic fertilization dose on SOC changes could not be derived from existing meta‐analytical studies, we included a negative feedback mechanism in the upscaling procedure allowing for higher fertilizer induced SOC changes in unfertilized or less fertilized soils.

#### 4.1.2 Impacts of climate

Based on our results there are no clear trends between climatic and management effects on SOC. Reduced tillage and organic fertilization were found to increase SOC along subtropical>temperate>tropical zones. For increased crop diversity, aSOC changes were greater in tropical>subtropical>temperate zones. Inorganic fertilization effects were most pronounced in subtropical>tropical>temperate climates. Due to a lack of data, it was not possible to make a climatic distinction for crop residue incorporation.

When comparing our results with the literature, we find certain discrepancies. Smith et al. (2008) estimated higher average SOC changes following reduced tillage and residue management in tropical moist, followed by tropical dry, temperate moist, and temperate dry regions. Data on tillage impacts in tropical regions were limited in their study, however, leading to potential uncertainties in their estimates for tropical zones (Smith et al., 2008). In contrast, Luo et al. (2010) suggested that the effect on SOC changes following reduced tillage was minimized in humid environments potentially due to faster decomposition of organic matter in surface layers, lowering the net impact of reduced tillage on SOC changes. Correspondingly, several field studies found that reduced tillage had more positive effects on SOC changes in dry compared to wet regions, potentially due to reduced SOC mineralization under dry conditions (Blanco‐Moure et al., 2013; Dimassi et al., 2014). Moreover, Cooper et al. (2016) found that the adoption of reduced till led to increased crop yields in Mediterranean systems, while the yields decreased in humid continental and humid oceanic regions under reduced tillage regimes. Besides reduced mineralization, the positive effects of reduced till on SOC under dryer climatic conditions might be linked to an increase in aboveground C inputs via enhanced crop production and residues remaining at the soil surface. Virto et al. (2012) found that about 30% of the variability of SOC stock changes under reduced tillage was attributed to differences in crop C inputs. Increased C inputs via organic matter application were also found to have more positive effects on SOC in temperate warm compared to temperate cool and tropical climates (Maillard & Angers, 2014). However, the authors noticed that tropical zones received overall lower manure C inputs compared to temperate regions which might explain the difference. Similarly, Han et al. (2016) found overall higher absolute SOC responses in temperate compared to tropical climates following improved fertilization. However, the opposite trend was found when the authors calculated the relative (percent) SOC changes. Han et al. (2016) suggested that the different effects on SOC could be attributed to differences in field study durations and initial SOC levels that varied per climate zone. In fact, it is likely that SOC changes per climate zone and management provided by our meta‐studies are biased due to the experimental subsets that differ in properties. The most relevant factors identified by meta‐studies included in our review were study duration, sampling depth, initial SOC levels and quality, and type of aboveground C inputs. Our results are likely to be sensitive to variations in these parameters.

#### 4.1.3 Impacts of other factors

*Study duration*: We found a large diversity of field study durations included in meta‐studies and systematic reviews. While some authors included studies with durations of less than 5 years (Jian et al., 2020; Ogle et al., 2005; Xu, Sieverding, et al., 2019), others included studies with minimum durations of more than 5 years (Angers & Eriksen‐Hamel, 2008; Virto et al., 2012; West & Post, 2002). Haddaway et al. (2017) used stricter inclusion criteria by selecting field studies that ran more than 10 years. Using stricter inclusion criteria leads to less variation in study durations between subgroups and, therefore, more robust estimates on SOC changes.

*Sampling depth*: Meta‐studies on increased fertilization and cover cropping often focus on average SOC changes in the top 20 cm (Han et al., 2016; King & Blesh, 2018; Poeplau & Don, 2015), while tillage studies mostly focus on the top 30‐cm depth (Haddaway et al., 2017; Meurer et al., 2018; Virto et al., 2012). The overall deeper sampling depths for tillage studies stem from the fact that tillage effects on SOC have been widely attributed to a redistribution of carbon in the plough layer, which is usually up to 30 cm. However, as tillage was often applied as co‐management in other meta‐studies such as those on cover cropping (King & Blesh, 2018; Poeplau & Don, 2015), it is, therefore, likely that the estimates on cover cropping in the top 20 cm are underestimated because one‐third of the cover crop effect could have been missed, as stressed by Poeplau and Don (2015).

*Soil texture*: Although soil texture is known to play an important role in stabilizing SOC (Virto et al., 2012), the findings on interactions between soil texture and SOC responses were often inconsistent among meta‐studies as also found in the synthesis of reviews by Bolinder et al. (2020). Besides this, we could not distinguish SOC changes in response to different soil texture and climatic zones as most meta‐studies did not provide separate estimates per management‐soil‐climate combination which hampered to create an additional stratification level for SOC responses in different soil types.

*Manure quality*: Furthermore, manure quality can affect the SOC sequestration rate (Berti et al., 2016). The included meta‐studies which provided data on liquid and solid manure, e.g., Maillard and Angers (2014) could, however, not evaluate the effects of solid and liquid manure on SOC stock changes due to the limited field study data on liquid manure. The meta‐study by Zavattaro et al. (2017) found, however, that the C sequestration potential from cattle farmyard manure was almost twice as high compared to a liquid slurry (see Table S8).

#### 4.1.4 Uncertainties in estimated SOC stock changes

The reliability of the calculated SOC stock changes is affected by many factors. One factor is the used bulk density to approximate stocks from concentrations. In the majority of the studies, bulk density was measured, while in only two studies, i.e., Haddaway et al. (2017) and Han et al. (2016), it was estimated. Unfortunately, we were not able to use pedo‐transfer functions to estimate bulk density from reported SOC concentrations (for an overview of various equations: see Gross and Glaser, (2021)) since original field study data from the relevant meta‐studies were rarely provided. The uncertainty induced using an estimated bulk density is likely not more than 15% in the study by Han et al. (2016), where we used an average bulk density of 1400 kg m^−3^, considering a global range of approximately 1200–1600 kg m^−3^. The uncertainty in the results of our study based on the tillage experiments reported by Haddaway et al. (2017) is likely even less since we were using spatially explicit and soil layer‐dependent bulk density estimates.

The impact of the large variability of field study data (i.e., differing residue management, crop rotation sequences, fertilizer management, etc.) in the various experiments on SOC stock changes is likely as significant as the uncertainty induced by the variation in bulk densities. This is illustrated by our results from the meta‐study of Haddaway et al. (2017) on tillage impacts compared to the original results (Figure S2). This study provided SOC stock changes, based on measured bulk densities, for part of the data, but also provided information on SOC concentration changes with coordinates of the original field‐study data for all data. This allowed us to convert SOC concentration to stock changes using spatially explicit and soil layer‐dependent bulk density estimates from SoilGrids (Hengl et al., 2014). The results (Figure S2) show that the estimated average stock changes from concentration data derived in this study are comparable to the measured SOC stock changes in the part of the data provided by Haddaway et al. (2017), but differences up to 15% do occur. The increased sample size in estimated stocks (275 pairwise tillage comparisons) compared to measured stocks only (127 pairwise tillage comparisons) leads to smaller variances and, therefore, more robust effect size estimates. Using fixed bulk densities, we also assumed that the overall effect of management on bulk density was negligible. Certain studies reported that reduced tillage can have positive effects on bulk density (Chatterjee & Lal, 2009; Dimassi et al., 2013), resulting in a potential overestimation in the derived SOC changes when using fixed bulk densities. Meurer et al. (2018) thus used the Equivalent Soil Mass (ESM) approach (Ellert & Bettany, 1995) to account for a decrease in bulk density due to less compaction while using data from tillage experiments by Haddaway et al. (2017). However, our results on stock estimates, based on the same Haddaway data and using a fixed bulk density approach, were not systematically different from those by Meurer et al. (2018). In contrast, the increased input of organic matter as compost can have negative effects on bulk density, but this has only been reported for exceptional cases with high doses applied over long time periods (Aguilera et al., 2013). The impacts of cover crop introduction and N addition on bulk density were found to be negligible (Lu et al., 2011; Poeplau & Don, 2015). In summary, most studies included measured data on bulk density, and impact of the variation and change in bulk densities on estimated SOC stock changes is limited.

Finally, our approach of using inverse variance weighting to summarize the meta‐study results comes with uncertainties in the estimated stock changes. Other than collecting data on all individual field study experiments from related meta‐studies to calculate the effect sizes, we decided to directly summarize the findings of the meta‐analyses to highlight the overall impact of management‐climate combinations on C sequestration potentials. For this, we used the inverse variance weighting method proposed by Tang et al. (2013) and as a consequence, we could not account for non‐independence as multiple meta‐analyses might include the same field study experiments potentially leading to biased variance estimates (Nakagawa et al., 2017; Tang et al., 2013). This, however, does not invalidate our calculation of the effect sizes itself, but potentially resulted in underestimating the variances of the meta‐analyses results (Tang et al., 2013). Due to a lack of data on the number of overlapping field studies from meta‐studies, we could not correct for this bias in variance estimates, e.g., by including an inflation factor based on the number of overlapping field studies as proposed by Tang et al. (2013).

### 4.2 Upscaling of results to the global scale

#### 4.2.1 Global‐scale SOC sequestration potential

In our study, we estimated the potential C sequestration in agricultural soils by assessing the areas where the measure can be applied, correcting for the areas where it has already been applied. The latter correction has not been applied by other global upscaling approaches, e.g., Zomer et al. (2017), Smith et al. (2008). With respect to replacing inorganic with organic fertilizer, we accounted for the fact that only the manure that is not currently recycled is available for the use on cropland. The C sequestration potential of manure currently applied to grassland soils has not been addressed in our study but might offer additional C storage potential when applied on croplands instead.

The fact that the comparison of organic farming versus conventional farming is mostly a movement of C from one site to another is often ignored in studies assessing the potential SOC sequestration of organic manure application (Leifeld et al., 2013). Our data indicate that <10% of the excreted manure is burned or wasted rather than recycled, which is in line with data presented by Uwizeye et al. (2020) for nitrogen. It strongly limits the potential of this method for SOC sequestration.

To increase SOC sequestration, we highlight the areas with large yield gaps have a high potential, which applies to many soils depleted in nutrients and organic matter, such as in sub‐Saharan Africa, South Asia, and subtropical and West Asia, as well as, further focusing on temperate soils where low SOC values may limit crop production (Oldfield et al., 2019). In these areas, there is the potential to raise food production by 42–70%, and to eventually even double it (Lobell et al., 2009; Mueller et al., 2012; see also www.yieldgap.org), which would largely enhance the crop residue input and subsequent C sequestration. Potential for measures reducing C decomposition via transition to minimal or no till systems apply for USA, Europe, and part of Northern Africa. Opportunities for C sequestration thus vary across regions given the local management and biophysical site conditions (Amelung et al., 2020) which control decomposition rates as well as net C inputs. The highest potential is found in regions with low crop production, low initial soil organic C levels, and in regions where livestock manure and crop residues are removed rather than applied to the soil. Practices that retain and increase SOC are well established and can increase the annual C stock between 440 and 683 Mton C yr^−1^ as shown from our analysis for cropland in non‐peaty soils, thereby assuming maximum complementarity among measures taken.

Recently, Fuss et al. (2018) summarized 22 studies with global potentials for soil carbon sequestration due to measures that increase carbon inputs and decrease carbon losses (mostly through decreased soil disturbance). All estimates are calculated in terms of a per‐area sequestration potential, whereas most of the variation was related to different assumptions on the areas available (i.e., area of managed cropland, grassland, degraded land) and the type of practices applied. For individual practices, the technical potentials for croplands vary from 1.47 to 2.93 Gt CO_2_ yr^−1^ (or 0.41–0.80 Gt C yr^−1^). Our estimate is strongly data‐driven and accounts for the current state of agricultural management, and hence, the potential is lower than the previous estimates. It also shows that the potential to sequester C in soil differs strongly from one region to another due to variations in the gaps between current and potential SOC levels. Our analysis supports the conclusion of Amelung et al. (2020) that a coordinated effort is required to identify effective measures adapted to the specific site properties controlling the potential to retain carbon. This requires spatially explicit and detailed insights on yield gaps, organic carbon levels, and the agronomic practices that are currently applied.

#### 4.2.2 Uncertainties in global scale SOC sequestration estimates

Even though our study follows a straightforward method for global estimation of potential C sequestration, potential errors might arise from the quality of the underlying meta‐analytical models, the assumptions around the potential area where agronomic measures can be applied as well as the system boundaries defined (e.g., by the total volume of C‐manure available and the assumption of an equilibrium status for soil organic matter in arable soils). Where the Monte Carlo approach accounted for the uncertainty related to the meta‐analytical models used, and total areas and C manure inputs were checked by global estimates from supporting papers, an in‐depth analysis of the error propagation of all data sources along the upscaling procedure has not been done since uncertainties on national statistics and open data sources are largely unknown and often not available. Crop land areas, as well as areas for application of C mitigating measures, have been derived from national statistics, ignoring the spatial variability in soil properties as well as management practices at farm and field level. New innovative downscaling procedures using remote sensing techniques will therefore reduce the uncertainty related to spatial allocation procedures (Amelung et al., 2020), in particular when ensemble methods are used to reduce error propagation (Van Looy et al., 2017). Similarly, high‐resolution soil property maps (Poggio et al., 2021; Van Looy et al., 2017) in combination with monthly weather data might help to underpin the actual soil C stocks, as well as decomposition rates, allowing more robust estimates of the *actual* C sequestration potential.

The meta‐analytical data on management impacts in tropical climates were systematically underrepresented due to fewer field study experiments performed in tropical climates as compared to temperate and sub‐tropical regions (Maillard & Angers, 2014; Poeplau & Don, 2015; Virto et al., 2012). Especially data on tillage impacts were heavily skewed toward field experiments performed in North America and Europe and, therefore, mostly representing impacts in temperate regions. To still obtain an overall effect estimate for tropical regions in the upscaling, we thus combined the effect sizes from all climate zones using the weighted average estimate. Considering that several meta‐studies on fertilization effects found that soils in tropical climates had overall lower potentials to store SOC than soils in temperate climates (Gross & Glaser, 2021; Ladha et al., 2011; Maillard & Angers, 2014), tillage impacts are likely overestimated in the upscaling for tropical regions. This hypothesis should, however, be substantiated by further measurements in tropical climates as the climatic effects on SOC sequestration remain rather inconsistent among meta‐studies as discussed in section 4.1.2.

In our assessment of the maximum amount of recyclable animal C‐manure, we used an average humification coefficient of 50%, being the percentage that is not decomposed within one year. In literature, this value varies mostly between 0.30 and 0.75 (Veeken et al., 2017), depending on animal and manure type, feeding strategy, and pre‐processing techniques applied (Berti et al., 2016; Zavattaro et al., 2017). By choosing a humification coefficient, our estimation of the maximum possible C increase (used as cut‐off value) is rather optimistic, as it refers to the percentage that is not decomposed within one year and using this value only holds when annual C inputs will remain equal in coming decades. When this is not the case, the C‐retention coefficient will be lower as shown by Maillard and Angers (2014) who derived a C‐retention coefficient of 12% for animal manure over 18 years of continued manure application. Poulton et al. (2018) found an increase of 0.7–1.0 t C ha^−1^yr^−1^ in the first 20 years in the Hoosfield barley and Broadbalk wheat experiment at Rothamsted at an annual manure application near 3.2 t organic C ha^−1^ yr^−1^, implying a C retention coefficient near 25% in this period. However, after 80–100 years, the average increase in both experiments was 0.16 t C ha^−1^yr^−1^, which is only 5% of the C input. In summary, even the corrected amount of C sequestration by enhanced manure application of 30 Mton C yr^−1^, being 20 times lower than the uncorrected amount application of 592 Mton C yr^−1^ (see Table 3) is a maximum estimate and might be twice as low when manuring is not continued.

For future work, the accuracy of our estimate on the maximum C sequestration potential from manure could be improved by accounting for variations in manure types and related humification coefficients (Veeken et al., 2017). For this, meta‐analytical data which evaluate the effects of solid and liquid manure on SOC stock changes would be required which was missing in our case, e.g. Maillard and Angers (2014) could not evaluate the effects of solid and liquid manure on SOC stock changes due to limited field data. However, since the manure excess is rather limited (most of the manure is already recycled, Zhang et al. (2017), its impact on global C sequestration potentials per climate‐country region is assumed to be rather small.

## 5 CONCLUSION

Based on our integrative analysis of global meta‐studies on soil organic carbon (SOC), we found that the overall impacts of improved cropland management range on an average from 0.2 to 0.6 t C ha^−1^ yr^−1^ over a timeframe of 20 years. The estimated global scale SOC sequestration potential is on average near 0.56 Gt C yr^−1^ (assuming maximum synergy among measures taken) being on the lower end of the previously estimated range of 0.1–2 Gt C yr^−1^. While the increased use of organic or combined fertilizers in site applications showed (much) higher impacts on SOC changes than soil (tillage) and crop management, the global scale potential is (much) lower than no or reduced tillage and increased crop diversity due to the fact that the availability of organic fertilizer (manure) is limited. In fact, the actual potential to increase SOC by increased manure application to arable land was only about 5% of the potential amount that is estimated by simply upscaling results from meta‐analytical studies. This illustrates the need for proper upscaling procedures (i.e., by combining spatial allocation procedures with high‐resolution soil property maps and remote sensing data) and the fact that many global scale quantifications are likely overestimations.

Global variation in potential C sequestration varies not only with climate and cropping systems but also with the current management practices being applied. High potential for improved fertilization, as well manure application, occurs in the southern part of the hemisphere, whereas reduced tillage practices can be applied in almost all intensive agricultural systems. Crop diversification is another general guideline that is broadly applicable.

The impact of different climate zones on management and SOC changes remains difficult to assess. It is likely that the differences in SOC found here are the result of a combination of factors that differ per management and climate zone. For instance, reduced till in sub‐tropical climates can have more pronounced positive impacts on SOC due to (a) lower initial SOC stocks, (b) increased crop yields leading to higher crop C inputs, and (c) less SOC mineralization. We, therefore, recommend making a distinction between climate and management when estimating global carbon sequestration potentials.

## 6 MATERIALS AND CORRESPONDENCE

Correspondence and requests for materials should be addressed to Lessmann.

## AUTHOR CONTRIBUTIONS

Lessmann and Ros: Collection and analysis of data. Ros, De Vries: Derivation of upscaling procedure. Lessmann, Ros, Young, De Vries: Drafting and critical revision of manuscript. Lessmann, Ros, Young, De Vries: Approval of published version and accountability of the work.

## COMPETING INTERESTS

The authors declare no competing interests.

### DATA AVAILABILITY STATEMENT

The data that support the findings of this study are available in https://github.com/AgroCares/globalcarbonpotential

## Supporting information

Data S1

Dats S2
