# Supplementary material for: Global variation in soil carbon sequestration potential through improved cropland management
Source: Glob Chang Biol. 2021 Nov 12;28(3):1162–77. doi: 10.1111/gcb.15954 (PMC9299007; doi:10.1111/gcb.15954)
Supplement: Supplementary file 1 — Data S1 [file GCB-28-1162-s002.docx]

**Global variation in soil carbon sequestration potential through improved cropland management**

M. Lessmann^1^, G.H. Ros^2^, M.D. Young^2^, and W. de Vries^2^

^1^Wageningen University and Research, Land Use Planning Group, P.O. Box 47, 6700 AA Wageningen, The Netherlands

^2^Wageningen University and Research, Environmental Systems Analysis Group, PO Box 47, NL-6700 AA Wageningen, the Netherlands

**Supplementary information on datasets that are used for the Upscaling Approach**

**Climate zone**. We selected climatic zones given the KÖPPEN-GEIGER climate classification map of the world (Peel et al., 2007) accessed via <http://koeppen-geiger.vu-wien.ac.at/present.htm>. Given the availability of meta-analysis results, we simplified the map into to four groups tropical, sub-tropical, temperate, and other climates (see Table S5).

**Land use**. We used data on area and yield for 41 crops from a Spatial Production Allocation Model (SPAM) (IFPRI et al., 2016; Wood-Sichra et al., 2016) with a resolution of 5 arc minute globally for the year 2005 (SPAM includes data for 2000 and 2005). We then rescaled the cropland area to the year 2018 by multiplying the crop land area of 2005 from SPAM with a country based correction factor, accounting for the increase in national cropland area from 2005 to 2018 from FAOSTAT (FAO, 2020a). The fraction area cultivated by cereals per country was used as variable for the cropland rotation with or without cereals (as being used for the development of the global tillage map).

**Tillage** **practices**. Insight in the current application of tillage and the potentially suitable area to apply no tillage (Conservation Agriculture) was derived from a global tillage system dataset (Porwollik et al., 2019). Data set and source code are accessible via an open-data repository via <ftp://datapub.gfz-potsdam.de/download/10.5880.PIK.2019.011/>. This dataset presents six tillage systems for 42 crop types (as included in the SPAM model) and potentially suitable Conservation Agriculture area as variables for gridded crop-specific cropland areas at the same resolution as SPAM, i.e. 5 arcmin. We aggregated the results to three groups

- - high intensity tillage (TILL_H): conventional annual tillage and rotational tillage
  - medium intensity tillage (TILL_M): traditional annual tillage, traditional rotational tillage and reduced tillage
  - no tillage (TILL_L): Conservation Agriculture

**Fertilization**. Spatially explicit data on global N fertilizer application rates were derived at a 0.5x 0.5 degree latitude–longitude resolution (Lu & Tian, 2016), on global N manure production and application rates on cropland at a 5 arcmin resolution (Zhang et al., 2017), being the same resolution as SPAM, and on global N manure application rates on grassland and N grazing at 5 arcmin resolution from Xu et al. (2019). Because the sequestration potential varies by the N-status (and related organic matter decomposition in the soil) we differentiated between five categories in N fertilization as given in Table S4. The total N input is the combination of inorganic and organic N input.

**Crop residues**. From the FAO databases (FAO, 2020c)(FAO, 2020b) we retrieved estimates of the total carbon lost via burning of crop residues as well as the total carbon entering the soil via aboveground crop residues. We distinguished the two following categories:

- BURN: the area of land where burning of crop residues occurs and where more than 50% of the total harvested area (per country) is grown with cereals (in that case there is also sufficient organic matter available for crop residue incorporation). This 50% is a flexible cut-off that is used as a rough indication of the availability of straw in the area.
- INC: the remaining part of the area where crop residues are incorporated in the soil or are left on the surface.

**Crop rotation**. The potential of extra C sequestration due to changes in crop rotations (more intensively rooting crops like cereals or introduction of catch crops in the rotation scheme) was assessed by distinguishing between the two following categories:

- CATCH: the area of cropland where no cereals are grown and where the percentage of area that is cultivated with cereals (on a national basis) is less than 50%. This latter value is a flexible cut-off value that can be changed if needed. In this area of land there is potential for additional carbon storage due to cultivation of cereals or the introduction of catch crops.
- NOCATH: the remaining part of the area where there is limited opportunity for additional carbon storage by the introduction of more cereals or catch crops in the rotation.

**Total soil organic carbon levels**. Total soil organic carbon levels per climate zone were derived from ISRIC data, being available on 250m grids: <https://files.isric.org/soilgrids/data/recent/>.

**Upscaling Approach**

The meta-analysis results were upscaled to global SOC sequestration potentials by multiplying the estimates per management and climate with the potential area where the practice could be applied. Here we made a distinction between the most important driving factors, including fertilization level, cropping system, climate zone (tropical, subtropical, temperate) and currently applied management. For increased fertilization, we distinguished between non, medium and highly fertilized systems where medium and highly fertilized croplands were further differentiated between systems with low and high animal manure inputs. The classification was based on combinations of N fertilizer and N manure inputs (Table S6). For the other management measures, we classified areas according to no, medium and high intensity tillage systems, crop rotations (without and with cereals/catch crops) and crop residue treatments (without and with potential for crop residue incorporation). When different meta-studies covered the same management-impact pair, we provide averages of the reported effect sizes, weighted by standard deviation as described in Young et al. (2020):

$\bar{x}=\frac{\sum\left( {x_{i}}/{\sigma_{i}^{2}} \right)}{\sum\left( 1/{\sigma_{i}^{2}} \right)}$ *(Eq. S1)* $\sigma_{\bar{x}}=\frac{1}{\sqrt{\sum(1/{\sigma_{i}^{2}})}}$ *(Eq. S2)*

Where*:* $\bar{x}$ *= weighted mean;* $\sigma_{\bar{x}}$ *= standard error of weighted mean;* $x_{i}$ *= individual mean from reported effect size and* $\sigma_{i}^{2}$ *= individual variance from reported effect size*

The weighted mean response and standard deviation for SOC due to a measure for different climate zones are summarized in Table 2. To link meta-analytical model derived SOC responses to the areas of agricultural land, we used the assumptions summarized in Table S6. The MA-model abbreviations match those listed in the main text in Table 1.

The total impact of additional manure addition on SOC sequestration was calculated as the difference between produced (excreted) N manure minus the N manure that is left on pasture by grazing minus the applied N manure on cropland and grassland, multiplied by the C-to-N ratio of manure and a fraction that remains after one year, according to:

C_potential_ = (N_production_ – N_application,cropland_-N_application,grassland_ -N_grazing_) x C/N x f_hum_ *(Eq. S3)*

With N_production_ and N_application,cropland_ from Zhang et al. (2017) and with N_application,grassland_ and N_grazing_ from Xu et al. (2019), assuming a C-to-N ratio of 10 and a humification coefficient of 50%.

**Supplementary References**

(IFPRI), I. F. P. R. I., (IIASA), I. I. for A. S. A., Foundation, T. B. and M. G., Intensification, U. F. the F. I. L. for C. R. on S., & Irrigation, U. F. the F. I. L. for S. S. (2016). *Global Spatially-Disaggregated Crop Production Statistics Data for 2005 Version 3.2* (I. F. P. R. Institute & I. I. for A. S. A. (IIASA) (eds.); V9 ed.). Harvard Dataverse. https://doi.org/doi/10.7910/DVN/DHXBJX

FAO. (2020a). *FAOSTAT, Agricultural Production, Crops Primary*. http://www.fao.org/faostat/en/#data/QC/visualize (Accessed: April 2020)

FAO. (2020b). *FAOSTAT Emissions Database, Agriculture, Burning - Crop Residues*. http://www.fao.org/faostat/en/#data/GB (Accessed: April-2020)

FAO. (2020c). *FAOSTAT Emissions Database, Agriculture – Crop Residues*. http://www.fao.org/faostat/en/#data/GA (Accessed:April 2020)

Lu, C., & Tian, H. (2016). Global nitrogen and phosphorus fertilizer use for agriculture production in the past half century: Shifted hot spots and nutrient imbalance. *Earth System Science Data Discussions*, *August*, 1–33. https://doi.org/10.5194/essd-2016-35

Peel, M. C., Finlayson, B. L., & Mcmahon, T. A. (2007). Hydrology and Earth System Sciences Updated world map of the Köppen-Geiger climate classification. In *Hydrol. Earth Syst. Sci* (Vol. 11). www.hydrol-earth-syst-sci.net/11/1633/2007/

Porwollik, V., Rolinski, S., Heinke, J., & Müller, C. (2019). Generating a global gridded tillage dataset. *Earth System Science Data Discussions*, *2015*, 1–28. https://doi.org/10.5194/essd-2018-152

Wood-Sichra, U., Joglekar, A. B., & You, L. (2016). Spatial Production Allocation Model (SPAM) 2005: Technical Documentation. *Harvest Choice*, 1–87. http://mapspam.info

Xu, R., Tian, H., Pan, S., Dangal, S. R. S., Chen, J., Chang, J., Lu, Y., Maria Skiba, U., Tubiello, F. N., & Zhang, B. (2019). Increased nitrogen enrichment and shifted patterns in the world’s grassland: 1860-2016. *Earth System Science Data*, *11*(1), 175–187. https://doi.org/10.5194/essd-11-175-2019

Young, M.D., De Vries, W., & Ros, G. H. (2019). *INSPIRATION bulletin: Integrated use of meta-analytical data to identify management trade-offs on crop growth, soil quality and environmental quality in agriculture* (Vol. 2, Issue September).

Young, Madaline D., Ros, G. H., & de Vries, W. (2020). A decision support framework assessing management impacts on crop yield, soil carbon changes and nitrogen losses to the environment. *European Journal of Soil Science*, *July*. https://doi.org/10.1111/ejss.13024

Zhang, B., Tian, H., Lu, C., Dangal, S. R. S., Yang, J., & Pan, S. (2017). Manure nitrogen production and application in cropland and rangeland during 1860 - 2014: A 5-minute gridded global data set for Earth system modeling. *Earth System Science Data Discussions*, 1–35. https://doi.org/10.5194/essd-2017-11
