# Supplementary material for: Global variation in soil carbon sequestration potential through improved cropland management
Source: Glob Chang Biol. 2021 Nov 12;28(3):1162–77. doi: 10.1111/gcb.15954 (PMC9299007; doi:10.1111/gcb.15954)
Supplement: Supplementary file 2 — Dats S2 [file GCB-28-1162-s001.docx]

**Global variation in soil carbon sequestration potential through improved cropland management**

M. Lessmann^1^, G.H. Ros^2^, M.D. Young^2^, and W. de Vries^2^

^1^Wageningen University and Research, Land Use Planning Group, P.O. Box 47, 6700 AA Wageningen, The Netherlands

^2^Wageningen University and Research, Environmental Systems Analysis Group, PO Box 47, NL-6700 AA Wageningen, the Netherlands

**Supplementary Tables**

**Table S1**: Overview of search strings organized by study type, subject, intervention and response

| **Search topic** | **Terms** |
| --- | --- |
| Study type | ‘meta-analysis’, ‘meta-analyses’, ‘review’ |
| Subject | ‘soil*’, ‘agricult*’, ‘arable’, ‘farm’, ‘agronom*’,’cult*’ |
| Intervention | ‘nutrient*’,‘fertilizer’, ‘organic’, ‘manure’, ‘slurry’, '4R', ‘mineral’, ‘inorganic’, 'nutrient management', ‘till*’, 'no till’, 'reduced till', 'conventional till’, 'soil management', 'cover crop', ‘crop’, 'rotation', 'long-term cultivation', 'residue', ’incorp*’, ’crop management' |
| Response | ‘SOC’, ‘soil organic carbon’, ‘soil C’, and ‘carbon sequest*. |

**Table S2:** Overview of included meta-studies for different measures, with original treatment and control groups descriptions and classified into climate zones, management measures and intervention categories.

Increased inorganic fertilization (M1): inorganic fertilizer - no fertilizer (IF-NF); Increased organic matter input (M2): Combined Fertilizer relative to Inorganic Fertilizer (COF-IF), Combined Fertilizer relative to No Fertilizer (COF-NF), Combined Straw + fertilizer relative to No Fertilizer (CRF-NF), Organic Fertilizer relative to Inorganic Fertilizer (OF-IF), Organic Fertilizer relative to No Fertilizer (OF-NF); Decreased tillage (M3): No-Till relative to High Intensity-Till (NT-HT), No-Till relative to Intermediate Intensity-Till (NT-IT) and Intermediate Intensity-Till relative to High Intensity-Till (IT-HT); Increased crop diversity (M4): crop diversification vs. monocultures (C), Crop rotation + cover crops (CC) and perennial crop rotation (CCP); Crop residue incorporation (M5): crop residue incorporation vs. removal (CRES).

| **Study number** | **Reference number** | **Authors and publication year** | **Regional coverage** | **Climate zone** | **Original treatment and control description** | **Intervention** | **Measure** |
| --- | --- | --- | --- | --- | --- | --- | --- |
| 1 | 1a | Aguilera et al. (2013) | Mediterranean | subtr | Organic amendments including compost + manure + industrial waste, no mineral fertilizer applied) vs. conventional management | OF-IF | M2 |
| 1 | 1b | Aguilera et al. (2013) | Mediterranean | subtr | Cover crops substituting bare fallows | CC | M4 |
| 1 | 1c | Aguilera et al. (2013) | Mediterranean | subtr | No tillage vs. conventional tillage | NT-HT | M3 |
| 1 | 1d | Aguilera et al. (2013) | Mediterranean | subtr | Reduced tillage vs. conventional tillage | IT-HT | M3 |
| 2 | 2a | Angers & Eriksen-Hamel (2008) | Global | temp | No till compared with full inversion till | NT-HT | M3 |
| 3 | 3a | Cooper et al. (2016) | Global | temp | Reduced tillage relative to deep inversion tillage | IT-HT | M3 |
| 4 | 4a | Haddaway et al. (2017) | Koeppen-Geiger zones: Cfa, Csa, Csb | subtr | Intermediate intensity till versus high intensity till (IT-HT) | IT-HT | M3 |
| 4 | 4b | Haddaway et al. (2017) | Koeppen-Geiger zones: Cfb, Dfa, Dfb, Dfc | temp | Intermediate intensity till versus high intensity till (IT-HT) | IT-HT | M3 |
| 4 | 4c | Haddaway et al. (2017) | Koeppen-Geiger zones: Cfa, Csa, Csb | subtr | No till versus high intensity till (NT-HT) | NT-HT | M3 |
| 4 | 4d | Haddaway et al. (2017) | Koeppen-Geiger zones: Cfb, Dfa, Dfb, Dfc | temp | No till versus high intensity till (NT-HT) | NT-HT | M3 |
| 4 | 4e | Haddaway et al. (2017) | Koeppen-Geiger zones: Cfa, Csa, Csb | subtr | No till versus intermediate intensity till (NT-IT) | NT-IT | M3 |
| 4 | 4f | Haddaway et al. (2017) | Koeppen-Geiger zones: Cfb, Dfa, Dfb, Dfc | temp | No till versus intermediate intensity till (NT-IT) | NT-IT | M3 |
| 5 | 5a | Han et al. (2016) | Tropical | trop | Chemical fertilizer N,P,K vs. no fertilizer | IF-NF | M1 |
| 5 | 5b | Han et al. (2016) | Warm temperate | subtr | Chemical fertilizer N,P,K vs. no fertilizer | IF-NF | M1 |
| 5 | 5c | Han et al. (2016) | Cool temperate | temp | Chemical fertilizer N,P,K vs. no fertilizer | IF-NF | M1 |
| 5 | 5d | Han et al. (2016) | Tropical | trop | Manure and chemical fertilizers relative to no fertilization | COF-NF | M2 |
| 5 | 5e | Han et al. (2016) | Warm temperate | subtr | Manure and chemical fertilizers relative to no fertilization | COF-NF | M2 |
| 5 | 5f | Han et al. (2016) | Cool temperate | temp | Manure and chemical fertilizers relative to no fertilization | COF-NF | M2 |
| 5 | 5g | Han et al. (2016) | Tropical | trop | Straw return and application of chemical fertilizers relative to no fertilization | CRF-NF | M2 |
| 5 | 5h | Han et al. (2016) | Warm temperate | subtr | Straw return and application of chemical fertilizers relative to no fertilization | CRF-NF | M2 |
| 5 | 5i | Han et al. (2016) | Cool temperate | temp | Straw return and application of chemical fertilizers relative to no fertilization | CRF-NF | M2 |
| 5 | 5j | Han et al. (2016) | Tropical | trop | Unbalanced Chemical fertilizer vs. no fertilizer | IF-NF | M1 |
| 5 | 5k | Han et al. (2016) | Warm temperate | subtr | Unbalanced Chemical fertilizer vs. no fertilizer | IF-NF | M1 |
| 5 | 5l | Han et al. (2016) | Cool temperate | temp | Unbalanced Chemical fertilizer vs. no fertilizer | IF-NF | M1 |
| 6 | 6a | Jian et al. (2020) | Tropical | trop | Cover crops vs. rotations without cover crops | CC | M4 |
| 6 | 6b | Jian et al. (2020) | Temperate | temp | Cover crops vs. rotations without cover crops | CC | M4 |
| 7 | 7a | King & Blesh (2018) | Global | temp | cover cropped rotation vs. grain only | CC | M4 |
| 7 | 7b | King & Blesh (2018) | Global | temp | perennial cropped rotation vs. grain only | CCP | M4 |
| 8 | 8a | Ladha et al. (2011) | Tropical | trop | Organic + Synthetic fertilizers relative to no fertilization | COF-NF | M2 |
| 8 | 8b | Ladha et al. (2011) | Humid subtropical | subtr | Organic + Synthetic fertilizers relative to no fertilization | COF-NF | M2 |
| 8 | 8c | Ladha et al. (2011) | Temperate | temp | Organic + Synthetic fertilizers relative to no fertilization | COF-NF | M2 |
| 8 | 8d | Ladha et al. (2011) | Tropical | trop | Organic fertilizer relative to no fertilization | OF-NF | M2 |
| 8 | 8e | Ladha et al. (2011) | Humid subtropical | subtr | Organic fertilizer relative to no fertilization | OF-NF | M2 |
| 8 | 8f | Ladha et al. (2011) | Temperate | temp | Organic fertilizer relative to no fertilization | OF-NF | M2 |
| 8 | 8g | Ladha et al. (2011) | Tropical | trop | Synthetic N addition relative to no fertilization | IF-NF | M1 |
| 8 | 8h | Ladha et al. (2011) | Humid subtropical | subtr | Synthetic N addition relative to no fertilization | IF-NF | M1 |
| 8 | 8i | Ladha et al. (2011) | Temperate | temp | Synthetic N addition relative to no fertilization | IF-NF | M1 |
| 9 | 9a | Lehtinen et al. (2014) | Europe | temp | Return of crop residues vs. Removal of crop residues | CRES | M5 |
| 10 | 10a | Lu et al. (2011) | Global | temp | N addition relative to no fertilization | IF-NF | M1 |
| 11 | 11a | Maillard & Angers (2014) | Tropical | trop | Manure relative to mineral fertilization | OF-IF | M2 |
| 11 | 11b | Maillard & Angers (2014) | Warm temperate | subtr | Manure relative to mineral fertilization | OF-IF | M2 |
| 11 | 11c | Maillard & Angers (2014) | Cool temperate | temp | Manure relative to mineral fertilization | OF-IF | M2 |
| 11 | 11d | Maillard & Angers (2014) | Tropical | trop | Manure relative to zero fertilization | OF-NF | M2 |
| 11 | 11e | Maillard & Angers (2014) | Warm temperate | subtr | Manure relative to zero fertilization | OF-NF | M2 |
| 11 | 11f | Maillard & Angers (2014) | Cool temperate | temp | Manure relative to zero fertilization | OF-NF | M2 |
| 12 | 12a | Mc Daniel et al. (2014) | Global | temp | Cover cropped rotations vs. monoculture | CC | M4 |
| 12 | 12b | Mc Daniel et al. (2014) | Global | temp | Crop rotations without cover crops | C | M4 |
| 13 | 13a | Meurer et al. (2018) | Boreo-temperate | temp | Intermediate intensity till versus high intensity till (IT-HT) | IT-HT | M3 |
| 13 | 13b | Meurer et al. (2018) | Boreo-temperate | temp | No till versus high intensity till (NT-HT) | NT-HT | M3 |
| 13 | 13c | Meurer et al. (2018) | Boreo-temperate | temp | No till versus intermediate intensity till (NT-IT) | NT-IT | M3 |
| 14 | 14a | Ogle et al. (2005) | Tropical moist | trop | No-till vs. conventional till | NT-HT | M3 |
| 14 | 14b | Ogle et al. (2005) | Tropical dry | trop | No-till vs. conventional till | NT-HT | M3 |
| 14 | 14c | Ogle et al. (2005) | Temperate dry | temp | No-till vs. conventional till | NT-HT | M3 |
| 14 | 14d | Ogle et al. (2005) | Temperate moist | temp | No-till vs. conventional till | NT-HT | M3 |
| 14 | 14e | Ogle et al. (2005) | Tropical moist | trop | Reduced tillage relative to conventional till | IT-HT | M3 |
| 14 | 14f | Ogle et al. (2005) | Tropical dry | trop | Reduced tillage relative to conventional till | IT-HT | M3 |
| 14 | 14g | Ogle et al. (2005) | Temperate moist | temp | Reduced tillage relative to conventional till | IT-HT | M3 |
| 14 | 14h | Ogle et al. (2005) | Temperate dry | temp | Reduced tillage relative to conventional till | IT-HT | M3 |
| 15 | 15a | Popleau & Don (2015) | Global | temp | Cover cropped rotations | CC | M4 |
| 16 | 16a | Spiegel et al. (2014) | Europe | temp | Cover crops/green manure vs. monoculture | CC | M4 |
| 16 | 16b | Spiegel et al. (2014) | Europe | temp | Mineral fertilizer application relative to no fertilization | IF-NF | M1 |
| 16 | 16c | Spiegel et al. (2014) | Europe | temp | Minimum and reduced non inversion tillage | IT-HT | M3 |
| 16 | 16d | Spiegel et al. (2014) | Europe | temp | No till vs. ploughing | NT-HT | M3 |
| 17 | 17a | Virto et al. (2012) | Global | temp | No till versus Inversion tillage | NT-HT | M3 |
| 18 | 18a | West & Post (2002) | Global | temp | No till versus conventional till | NT-HT | M3 |
| 18 | 18b | West & Post (2002) | Global | temp | Enhancement of rotation complexity vs. monoculture or bare fallows | CC | M4 |
| 19 | 19a | Xu et al. (2019) | Global | temp | Corn stover retention | CRES | M5 |
| 20 | 20a | Zavaratto et al. (2017) | Europe | temp | Bovine slurry + mineral fertilizer relative to mineral fertilization only | COF-IF | M2 |
| 20 | 20b | Zavaratto et al. (2017) | Europe | temp | Bovine Slurry application relative to mineral fertilization only | OF-IF | M2 |
| 20 | 20c | Zavaratto et al. (2017) | Europe | temp | Farmyard manure + mineral fertilizer relative to mineral fertilization only | COF-IF | M2 |
| 20 | 20d | Zavaratto et al. (2017) | Europe | temp | Farmyard manure application relative to mineral fertilization only | OF-IF | M2 |

**Table S3:** Overview on selected meta studies and reviews classified by management intervention and SOC changes. From the 20 studies, only 14 were used (studies given in bold) to derive absolute values on SOC stock changes, since the remaining studies only provided estimates on relative SOC changes with missing data on initial stocks.

Site-specific factors addressed by sub-group analysis or meta-regression to account for heterogeneity are distinguished by climate variables, soil variables and other factors such as study duration, sampling depth etc. Management classified into increased fertilization (M1) with inorganic fertilizer relative to no fertilizer application (IF-NF), increased organic fertilization (M2) including combined residue incorporation and inorganic fertilizer application relative to no fertilizer (CRF-NF), combined organic and inorganic fertilizer application relative to no fertilizer (COF-NF), organic fertilizer relative to no fertilizer application (OF-NF), combined organic and inorganic relative to inorganic fertilizer application (COF-IF), organic fertilizer relative to inorganic fertilizer (OF-IF). Reduced tillage (M3) is classified into no-till relative to high intensity-till (NT-HT), no-till relative to intermediate intensity-till (NT-IT) and intermediate intensity-till relative to high intensity-till (IT-HT). Increased crop diversity (M4) including increased crop rotations vs. monocultures (C), increased rotations plus cover crops (CC) and perennial cropped rotations (CCP). Crop residue incorporation (M5) including crop residue incorporation relative to residue removal (CRES).

| **No** | **Authors** | **Regional coverage** | **Management** | | | | | | | | | | | | | **SOC changes** | | | | | | | | **Site-specific factors** | | |
| --- | --- | --- | --- | --- | --- | --- | --- | --- | --- | --- | --- | --- | --- | --- | --- | --- | --- | --- | --- | --- | --- | --- | --- | --- | --- | --- |
|  |  |  | **M1** | **M2** | | | | | **M3** | | | **M4** | | | **M5** | **SOC stocks** | | | | **SOC contents** | | | | **Climate variables (continuous or categorical)** | **Soil variables (continuous or categorical)** | **Other factors** |
|  |  |  | IF-NF | CRF-NF | COF-NF | OF - NF | COF-IF | OF-IF | NT-HT | NT-IT | IT-HT | CCP | CC | C | CRES | ∆ SOC (ton ha^-1^ yr^-1^) | ∆ SOC (ton ha^-1^) | ∆ SOC (% yr^-1^) | ∆ SOC (%) | ∆ SOC (g kg-1 yr^-1^) | ∆ SOC (g kg^-1^) | ∆ SOC (% yr^-1^) | ∆ SOC (%) |  |  |  |
| **1** | **Aguilera et al. (2013)** | Mediterranean |  |  |  |  |  | **x** | **x** |  | **x** |  | **x** |  |  | **x** |  |  | **x** |  |  |  |  | - | - | management intensity; type of organic input; tillage; fertilizer application;  experimental approach |
| **2** | **Angers & Eriksen-Hamel (2008)** | Global |  |  |  |  |  |  | **x** |  |  |  |  |  |  |  | **x** |  |  |  |  |  |  | temperature; precipitation | USDA soil classification | experiment duration |
| **3** | **Cooper et al. (2016)** | Global |  |  |  |  |  |  |  |  | **x** |  |  |  |  |  | **x** |  |  |  |  |  |  | - | - | sampling depth |
| **4** | **Haddaway et al. (2017)** | Boreo-temperate regions |  |  |  |  |  |  | **x** | **x** | **x** |  |  |  |  |  | **x** |  |  |  | **x** |  |  | Köppen–Geiger climate classification | USDA soil classification; SOC reference | experiment duration; tillage depth; latitude |
| **5** | **Han et al. (2016)** | Global | **x** | **x** | **x** |  |  |  |  |  |  |  |  |  |  |  |  |  |  | **x** | **x** |  | **x** | cool temperate; warm temperate; tropical | - | fertilization application duration; C input |
| **6** | **Jian et al. (2020)** | Global |  |  |  |  |  |  |  |  |  |  | **x** |  |  | **x** |  |  | **x** |  |  |  |  | temperate; tropical; arid; snowy | soil texture classes: coarse, medium, fine | cover crop type; sampling depth; |
| **7** | **King & Blesh (2018)** | Global |  |  |  |  |  |  |  |  |  | **x** | **x** |  |  |  | **x** |  | **x** |  |  |  |  | latitude | clay ; SOC concentration; pH | elevation; experiment duration; sampling depth |
| 8 | Ladha et al. (2011) | Global | **x** |  | **x** | **x** |  |  |  |  |  |  |  |  |  |  |  |  |  |  |  |  | **x** | temperate; tropical; humid-subtropical | - | type of land use (flooded, flooded dryland, dryland) |
| 9 | Lehtinen et al. (2014) | Europe |  |  |  |  |  |  |  |  |  |  |  |  |  |  |  |  |  |  |  |  | **x** | Nemoral; Atlantic; Continental; Mediterranean | clay | experiment duration |
| **10** | **Lu et al. (2011)** | Global | **x** |  |  |  |  |  |  |  |  |  |  |  |  |  |  |  |  |  |  |  | **x** | precipitation; temperature; latitude | - | experiment duration; sampling depth; N application rate |
| **11** | **Maillard & Angers (2014)** | Global |  |  |  | **x** |  | **x** |  |  |  |  |  |  |  |  | **x** |  | **x** |  |  |  | **x** | cool temperate; warm temperate; tropical | clay; silt; initial SOC concentration | cumulative manure-C input; animal species; land use (annual, perennial, paddy) |
| 12 | Mc Daniel et al. (2014) | Global |  |  |  |  |  |  |  |  |  |  | **x** | **x** |  |  |  |  |  |  |  |  | **x** | precipitation; temperature | sand; clay | tillage practice; fertilizer rates; number of crops in rotation; experiment duration; sampling depth |
| **13** | **Meurer et al. (2018)** | Boreo-temperate regions |  |  |  |  |  |  | **x** | **x** | **x** |  |  |  |  | **x** | **x** |  | **x** |  |  |  |  | Köppen–Geiger climate classification | USDA soil classification | experiment duration; tillage depth; crop yields; cropping index |
| 14 | Ogle et al. (2005) | Global |  |  |  |  |  | **x** | **x** |  | **x** |  |  |  |  |  |  |  | **x** |  |  |  |  | temperate moist; temperate dry; tropical moist; tropical dry | - | sampling depth; experiment duration |
| **15** | **Popleau & Don (2015)** | Global |  |  |  |  |  |  |  |  |  |  | **x** |  |  | **x** |  |  |  |  |  |  |  | temperate; tropic | - | experiment duration; sampling depth; elevation |
| 16 | Spiegel et al. (2014) | Europe | **x** |  |  |  |  |  | **x** |  | **x** |  | **x** |  |  |  |  |  | **x** |  |  |  | **x** | Nemoral; Atlantic; Continental; Mediterranean | clay | experiment duration; sampling depth |
| **17** | **Virto et al. (2012)** | Global |  |  |  |  |  |  | **x** |  |  |  |  |  |  |  | **x** |  | **x** |  |  |  |  | precipitation; temperature; aridity | USDA soil classification | Crop C inputs |
| **18** | **West & Post (2002)** | Global |  |  |  |  |  |  | **x** |  |  |  | **x** |  |  | **x** | **x** |  |  |  |  |  |  | - | - | experiment duration |
| **19** | **Xu et al. (2019)** | Global |  |  |  |  |  |  |  |  |  |  |  |  | **x** | **x** |  |  |  |  |  |  |  | precipitation; temperature | USDA soil classification | sampling depth; reference stock; irrigation; rotation type; stover removal rate; tillage system ; study duration |
| 20 | Zavaratto et al. (2017) | Europe |  |  |  |  | **x** | **x** |  |  |  |  |  |  |  |  |  |  |  |  |  |  | **x** | Northern; Eastern; Western; Southern | light; medium; heavy textured | experiment duration; crop class; tillage depth |

**Table S4**. Categories used for upscaling fertilizer induced responses on soil organic carbon (SOC)

| **Category** | **Description** | **Total N input**  **(kg N / ha)** | **N input animal manure**  **(kg N / ha)** |
| --- | --- | --- | --- |
| NIFNOF | Non fertilized | < 40 |  |
| MIFNOF | medium fertilized, low animal N-input | < 100 | < 40 |
| MIFHOF | medium fertilized, high animal N-input | < 100 | ≥ 40 |
| HIFNOF | highly fertilized, low animal N-input | ≥ 100 | < 40 |
| HIFHOF | highly fertilized, high animal N-input | ≥ 100 | ≥ 40 |

**Table S5:** KOEPPEN GEIGER climate zone classification into tropical (trop), subtropical (subtr), temperate (temp) and other (oth)

| NR | GRID_CODE | GK_CODE | GK_GROEP |
| --- | --- | --- | --- |
| 1 | 11 | Af | trop |
| 2 | 12 | Am | trop |
| 3 | 13 | As | trop |
| 4 | 14 | Aw | trop |
| 5 | 21 | BWk | oth |
| 6 | 22 | BWh | oth |
| 7 | 26 | BSk | oth |
| 8 | 27 | BSh | oth |
| 9 | 31 | Cfa | subtr |
| 10 | 32 | Cfb | temp |
| 11 | 33 | Cfc | temp |
| 12 | 34 | Csa | subtr |
| 13 | 35 | Csb | subtr |
| 14 | 36 | Csc | subtr |
| 15 | 37 | Cwa | subtr |
| 16 | 38 | Cwb | subtr |
| 17 | 39 | Cwc | subtr |
| 18 | 41 | Dfa | temp |
| 19 | 42 | Dfb | temp |
| 20 | 43 | Dfc | temp |
| 21 | 44 | Dfd | temp |
| 22 | 45 | Dsa | subtr |
| 23 | 46 | Dsb | subtr |
| 24 | 47 | Dsc | subtr |
| 25 | 48 | Dsd | subtr |
| 26 | 49 | Dwa | oth |
| 27 | 50 | Dwb | oth |
| 28 | 51 | Dwc | oth |
| 29 | 52 | Dwd | oth |
| 30 | 61 | EF | oth |
| 31 | 62 | ET | oth |

**Table S6.** Assumptions made to link changes in SOC from meta-analytical models to the different categories of cropland available.

| **Measure** | **Category** | **MA model** | **Explanation** |
| --- | --- | --- | --- |
| M1 | NIFNOF | IF-NF | Adding N-fertilizer enhances C input |
|  | MIFNOF | 0.5 * IF-NF | In medium N-fertilized soils, impact is smaller, estimated on 50% |
|  | MIFHOF |  |  |
|  | HIFNOF | 0.0 * IF-NF | Soils well fertilized show no response in SOC |
|  | HIFHOF |  |  |
| M2 | NIFNOF | OF-NF | Adding organic manure on unfertilized soils enhances SOC |
|  | MIFNOF | COF-NF – IF-NF | Impact is only due to additional C added via manure |
|  | MIFHOF |  | Impact is only due to fertilizer-N effect |
|  | HIFNOF | 0.5 * MIFHOF | Impact is limited and only due to C via manure |
|  | HIFHOF | 0 | no addition impact for SOC |
| M3 | TILL_M | NT-IT | Changing intermediate to no till enhances SOC |
|  | TILL_H | NT-HT | Changing intensive to no till enhances SOC |
|  | TILL_L | 0 | In no till systems, there is no impact |
| M4 | CATCH | CC | Adding catch crops enhances C input |
|  | NOCATCH | 0 | When catch crops are present, no impact possible |
| M5 | BURN | CRES | Use crop residues to enhance SOC |
|  | INC | 0 | No additional impact for SOC |

**Table S7:** Impact of increased inorganic fertilization (M1) on SOC stock changes in absolute (ton ha^-1^ yr^-1^, ton ha^-1^) and relative terms (% yr^-1^, %) based on data from meta-studies. Management interventions classified into inorganic fertilizer relative to no fertilizer application (IF-NF). Climate zones are classified into temperate (temp), subtropical (subtr), tropical (trop)

| **Intervention** | **treatment – control** | **Climate zone** | **Ref no** | **∆ SOC over 20 years**  **in ton ha^-1^ yr^-1^ ± se** | **∆ SOC**  **in ton ha^-1^ yr^-1^ ± se** | **∆ SOC**  **in ton ha^-1^ ± se** | **∆ SOC**  **in % yr^-1^** | **∆ SOC**  **in % ± se** | **Average study duration in years [range]** | **Number of paired comparisons** | **Average sampling depth in m [range]** | **original effect size unit** | **Regional coverage** |
| --- | --- | --- | --- | --- | --- | --- | --- | --- | --- | --- | --- | --- | --- |
| IF-NF | balanced mineral (N,P,K) fertilizer - no fertilizer | trop | 5a | 0.17 ±0.05** | 0.29 ±0.08*** | 2.72±0.44* | 1.63±0.26* | 19.6±3.16 | 12 | 76 | [0-0.2] | concentration | tropical |
|  |  | subtr | 5b | 0.49 ±0.12** | 0.62±0.15*** | 3.86±0.59* | 1.01±0.14* | 16.1±2.30 | 16 | 89 | [0-0.2] | concentration | warm temperate |
|  |  | temp | 5c | 0.24 ±0.05** | 0.19 ±0.04*** | 6.89±1.91* | 0.46±0.06* | 11.9±1.66 | 26 | 97 | [0-0.2] | concentration | cool temperate |
|  | mineral (N) fertilizer - no fertilizer | trop | 8g | - | - | - | 0.80±0.10* | 16±2.04 | [6-158] | 83 | [0-0.6]^1^ | concentration | tropical |
|  |  | subtr | 8h | - | - | - | 0.55±0.06* | 11±1.28 | [6-158] | 160 | [0-0.6]^1^ | concentration | humid subtropical |
|  |  | temp | 8i | - | - | - | 0.15±0.04* | 3±0.77 | [6-158] | 195 | [0-0.6]^1^ | concentration | temperate |
|  | mineral (N) fertilizer - no fertilizer | temp | 10a | 0.06 ±0.01** | 0.06 ±0.01* | 1.25±0.14**** | 0.17±0.02* | 3.48±0.38 | [1-45] | 340 | [0-0.5] | concentration | Global^2^ |
|  | mineral (N) fertilizer - no fertilizer | temp | 16b | - | - | - | 0.15±0.06* | 3±1.17 | x | 40 | x | stock | Europe |
|  | unbalanced mineral (N,P,K) fertilizer - no fertilizer | trop | 5j | 0.17 ±0.07** | 0.28±0.11*** | 2.21±0.50* | 0.75±0.15* | 9±1.79 | 12 | 38 | [0-0.2] | concentration | tropical |
|  |  | subtr | 5k | 0.15 ±0.03** | 0.19±0.03*** | 2.30±0.24* | 0.61±0.07* | 9.7±1.07 | 16 | 119 | [0-0.2] | concentration | warm temperate |
|  |  | temp | 5l | 0.21 ±0.04** | 0.16±0.03*** | 3.16±0.54* | 0.42±0.11* | 11±2.81 | 26 | 50 | [0-0.2] | concentration | cool temperate |

*Mean changes estimated based on average study durations provided by meta-study. If no average duration was provided, a time horizon of 20 years was assumed

**Normalized annual SOC stock changes over 20 years

***Mean annual stock changes estimated from mean annual concentration changes

****Mean absolute stock changes calculated from relative stock changes

^X^ Categories as defined by the studies: Study duration: <5; 5–10; 11–20; >20; Sampling depth: 0–10; 11–30; >30

^1^Soil depths: 54% of data from 0-15cm, 39% from 0-30cm, 5% from 0-45cm, 3% from 0-60cm

^2^Most studies conducted in north America and Europe, thus classified as temperate

**Table S8:** Impact of increased organic fertilization (M2) on SOC stock changes in absolute (ton ha^-1^ yr^-1^, ton ha^-1^) and relative terms (% yr^-1^, %) based on data from meta-studies. Management interventions are classified into Combined Fertilizer relative to Inorganic Fertilizer (COF-IF), Combined Fertilizer relative to No Fertilizer (COF-NF), Combined Straw + fertilizer relative to No Fertilizer (CRF-NF), Organic Fertilizer relative to Inorganic Fertilizer (OF-IF), Organic Fertilizer relative to No Fertilizer (OF-NF). Climate zones are classified into temperate (temp), subtropical (subtr), tropical (trop)

| **Intervention** | **treatment - control** | **Climate zone** | **Ref no** | **∆ SOC over 20 years**  **in ton ha^-1^ yr^-1^ ± se** | **∆ SOC**  **in ton ha^-1^ yr^-1^ ± se** | **∆ SOC**  **in ton ha^-1^ ± se** | **∆ SOC**  **in % yr^-1^** | **∆ SOC**  **in % ± se** | **Average study duration in years [range]** | **Number of paired comparisons** | **Average sampling depth in m [range]** | **original effect size unit** | **Regional coverage** |
| --- | --- | --- | --- | --- | --- | --- | --- | --- | --- | --- | --- | --- | --- |
| COF-IF | Slurry + mineral (N) fertilizer - mineral (N) fertilizer | temp | 20a | - | - | - | 0.87±0.46* | 17.3±9.10 | x | 10 | x | concentration | Europe |
|  | Manure + mineral (N) fertilizer - mineral (N) fertilizer | temp | 20c | - | - | - | 1.65±0.26* | 33±5.12 | x | 54 | x | concentration | Europe |
| COF-NF | Manure + mineral (N,P,K) fertilizer - no fertilizer | trop | 5d | 0.51±0.05** | 0.85±0.08*** | 8.40±0.39* | 3.51±0.23* | 42.1±2.76 | 12 | 192 | [0-0.2] | concentration | tropical |
|  |  | subtr | 5e | 0.91±0.12** | 1.13±0.15*** | 10.70±0.63* | 2.51±0.16* | 40.2±2.60 | 16 | 209 | [0-0.2] | concentration | warm temperate |
|  |  | temp | 5f | 0.68±0.07** | 0.52±0.05*** | 9.60±0.79* | 1.11±0.09* | 28.8±2.27 | 26 | 251 | [0-0.2] | concentration | cool temperate |
|  | Organic + mineral (N) fertilizer - no fertilizer | trop | 8a | - | - | - | 1.50±0.22* | 30±4.34 | [6-158] | 41 | [0-0.6]^1^ | concentration | tropical |
|  |  | subtr | 8b | - | - | - | 1.95±0.20* | 39±4.08 | [6-158] | 66 | [0-0.6]^1^ | concentration | Humid subtropical |
|  |  | temp | 8c | - | - | - | 0.90±0.34 | 18±6.89 | [6-158] | 11 | [0-0.6]^1^ | concentration | temperate |
| CRF-NF | Straw + mineral (N,P,K) fertilizer - no fertilizer | trop | 5g | 0.71±0.15** | 1.18±0.24*** | 4.90±0.64* | 2.23±0.21* | 26.8±2.55 | 12 | 114 | [0-0.2] | concentration | tropical |
|  |  | subtr | 5h | 0.89±0.07** | 1.11±0.08*** | 5.99±0.36* | 1.09±0.07* | 17.4±1.07 | 16 | 324 | [0-0.2] | concentration | warm temperate |
|  |  | temp | 5i | 0.97±0.16** | 0.75±0.12*** | 5.54±0.51* | 0.69±0.08* | 18±2.04 | 26 | 182 | [0-0.2] | concentration | cool temperate |
| OF-IF | Manure - mineral (N) fertilizer | temp | 20d | - | - | - | 1.62±0.23* | 32.4±4.54 | x | 60 | X | concentration | Europe |
|  | Manure - mineral (N,P,K) fertilizer | trop | 11a | 0.19±0.05** | 0.22±0.05* | 3.87±0.97 | - | - | 18 | 33 | 0.26 | stock | tropical |
|  |  | subtr | 11b | 0.36±0.07** | 0.38±0.08* | 7.29±1.48 | - | - | 19 | 24 | 0.26 | stock | warm temperate |
|  |  | temp | 11c | 0.41±0.12** | 0.30±0.08* | 8.26±2.34 | - | - | 28 | 16 | 0.26 | stock | cool temperate |
|  | Organic - mineral (N,P,K) fertilizer | subtr | 1a | 0.41±0.13** | 1.31±0.43 | 8.12±2.69* | 3.79±0.85* | 23.5±5.26 | 6 | 37 | 0.23 | stock | Mediterranean |
|  | Slurry - mineral (N) fertilizer | temp | 20b | - | - | - | 0.88±0.11* | 17.5±2.25 | x | 23 | x | concentration | Europe |
| OF-NF | Manure - no fertilizer | trop | 11d | 0.32±0.07** | 0.37±0.08* | 6.33±1.41 | - | - | 17 | 21 | 0.26 | stock | tropical |
|  |  | subtr | 11e | 0.62±0.11** | 0.57±0.10* | 12.43±2.20 | - | - | 22 | 18 | 0.26 | stock | warm temperate |
|  |  | temp | 11f | 0.53±0.14** | 0.41±0.11* | 10.65±2.82 | - | - | 26 | 18 | 0.26 | stock | cool temperate |
|  | Organic - no fertilization | trop | 8d | - | - | - | 1.30±0.22* | 26±4.34 | [6-158] | 9 | [0-0.6]^3^ | concentration | tropical |
|  |  | subtr | 8e | - | - | - | 1.65±0.19* | 33±3.83 | [6-158] | 54 | [0-0.6]^3^ | concentration | Humid subtropical |
|  |  | temp | 8f | - | - | - | 1.15±0.60* | 23±11.99 | [6-158] | 25 | [0-0.6]^3^ | concentration | temperate |

*Mean changes estimated based on average study durations provided by meta-study. If no average duration was provided, a time horizon of 20 years was assumed

**Normalized annual SOC stock changes over 20 years

***Mean annual stock changes estimated from mean annual concentration changes

^X^ Categories as defined by the studies: Study duration: <5; 5–10; 11–20; >20; Sampling depth: 0–10; 11–30; >30

^1^ Soil depths: 54% of data from 0-15cm, 39% from 0-30cm, 5% from 0-45cm, 3% from 0-60cm

**Table S9:** Impact of reduced tillage (M3) on SOC stock changes in absolute (ton ha^-1^ yr^-1^, ton ha^-1^) and relative terms (% yr^-1^, %) based on data from meta-studies. *Management is classified into No-Till relative to High Intensity-Till (NT-HT), No-Till relative to Intermediate Intensity-Till (NT-IT) and Intermediate Intensity-Till relative to High Intensity-Till (IT-HT). Climate zones are classified into temperate (temp), subtropical (subtr), tropical (trop)*

| **Intervention** | **treatment - control** | **Climate zone** | **Ref no** | **∆ SOC over 20 years**  **in ton ha^-1^ yr^-1^ ± se** | **∆ SOC**  **in ton ha^-1^ yr^-1^ ± se** | **∆ SOC**  **in ton ha^-1^ ± se** | **∆ SOC**  **in % yr^-1^** | **∆ SOC**  **in % ± se** | **Average study duration in years [range]** | **Number of paired comparisons** | **Average sampling depth in m [range]** | **original effect size unit** | **Regional coverage** |
| --- | --- | --- | --- | --- | --- | --- | --- | --- | --- | --- | --- | --- | --- |
| IT-HT | Intermediate intensity till - high intensity till | subtr | 4a | 0.15±0.04** | 0.17±0.05*** | 3.07±0.86* | - | - | 18 | 38 | [0-0.3] | concentration/stock | Koeppen-Geiger zones: Cfa, Csa, Csb |
|  |  | temp | 4b | 0.10±0.03** | 0.11±0.03*** | 2.04±0.65* | - | - | 19 | 107 | [0-0.3] | concentration/stock | Koeppen-Geiger zones: Cfb, Dfa, Dfb, Dfc |
|  | Intermediate intensity till - high intensity till | temp | 13a | 0.18±0.08** | 0.22±0.10* | 3.5±1.66 | 0.38* | 6 | 16 | 27 | [0-0.3] | stock | Boreo-temperate |
|  | Noninversion till - high intensity till | temp | 16c | - | - | - | 0.30±0.06* | 6±1.15 | x | 410 | x | stock | Europe |
|  | Reduced till - high intensity till | subtr | 1d | 0.19±0.07** | 0.31±0.12 | 3.81±1.44* | 1.15±0.44* | 14.1±5.45 | 12 | 17 | 0.27 | stock | Mediterranean |
|  | Reduced till - high intensity till | temp | 3a | 0.07±0.02** | 0.07±0.02* | 1.43±0.34 | - | - | 20 | 184 | 0.25 | stock | Global^1^ |
|  | Reduced till – conventional till | trop | 14e | - | - | - | 0.80±0.25* | 16±5 | xx | NA | [0-0.3] | stock | tropical moist |
|  |  | trop | 14f | - | - | - | 0.50±0.25* | 10±5 | xx | NA | [0-0.3] | stock | tropical dry |
|  |  | temp | 14g | - | - | - | 0.45±0.20* | 9±4 | xx | NA | [0-0.3] | stock | temperate moist |
|  |  | temp | 14h | - | - | - | 0.15±0.15* | 3±3 | xx | NA | [0-0.3] | stock | temperate dry |
| NT-HT | No till - conventional till | subtr | 1c | 0.25±0.06** | 0.43±0.11 | 5.03±1.28* | 0.85±0.48* | 10±5.58 | 12 | 33 | 0.34 | stock | Mediterranean |
|  |  | trop | 14a | - | - | - | 1.15±0.25* | 23±5 | xx | NA | [0-0.3] | stock | tropical moist |
|  |  | trop | 14b | - | - | - | 0.85±0.25* | 17±5 | xx | NA | [0-0.3] | stock | tropical dry |
|  |  | temp | 14c | - | - | - | 0.50±0.15* | 10±3 | xx | NA | [0-0.3] | stock | temperate dry |
|  |  | temp | 14d | - | - | - | 0.80±0.10* | 16±2 | xx | NA | [0-0.3] | stock | temperate moist |
|  | No till - conventional till | temp | 18a | 0.40±0.10** | 0.57±0.14 | 7.1±1.75 | - | - | 14 | 79 | 0.22 | stock | Global^1^ |
|  | No till - full inversion till | temp | 2a | 0.25** | 0.31* | 4.9 | - | - | 16 | 24 | [0-0.3] | stock | Global^1^ |
|  | No till - high intensity till | subtr | 4c | 0.33±0.07** | 0.35±0.07*** | 6.64±1.34* | - | - | 19 | 65 | [0-0.3] | concentration/stock | Koeppen-Geiger zones: Cfa, Csa, Csb |
|  |  | temp | 4d | 0.13±0.04** | 0.15±0.04*** | 2.64±0.73* | - | - | 18 | 112 | [0-0.3] | concentration/stock | Koeppen-Geiger zones: Cfb, Dfa, Dfb, Dfc |
|  | No till - high intensity till | temp | 13b | 0.21±0.10** | 0.24±0.11* | 4.23±1.92 | 0.44* | 7 | 18 | 46 | [0-0.3] | stock | boreo-temperate |
|  | No till - inversion till | temp | 17a | 0.17** | 0.23 | 3.4 | 0.45±0.12* | 6.7±1.77 | 15 | 92 | [0-0.3] | stock | Global^1^ |
|  | No till - ploughing | temp | 16d | - | - | - | 0.35±0.12* | 7±2.32 | x | 131 | X | stock | Europe |
| NT-IT | No till - intermediate intensity till | subtr | 4e | 0.17±0.06** | 0.18±0.06*** | 3.48±1.12* | - | - | 19 | 79 | [0-0.3] | concentration/stock | Koeppen-Geiger zones: Cfa, Csa, Csb |
|  |  | temp | 4f | 0.07±0.03** | 0.08±0.04*** | 1.46±0.67* | - | - | 18 | 91 | [0-0.3] | concentration/stock | Koeppen-Geiger zones: Cfb, Dfa, Dfb, Dfc |
|  | No till - intermediate intensity till | temp | 13c | 0.11±0.08** | 0.12±0.08* | 2.22±1.51 | 0.11* | 2 | 18 | 25 | [0-0.3] | stock | Boreo-temperate |

*Mean changes estimated based on average study durations provided by meta-study. If no average duration was provided, a time horizon of 20 years was assumed

**Normalized annual SOC stock changes over 20 years

***Mean annual stock changes estimated from mean annual concentration changes based on in-depth analysis of field study data provided by Haddaway et al. (2017)

^x^ Categories as defined by the studies: Study duration: <5; 5–10; 11–20; >20; Sampling depth: 0–10; 11–30; >30

^xx^ Large range of study durations given from 2-100 years, not possible to give separate estimates per sub-group

^1^Most data from global temperate regions (e.g. north America and or Europe), therefore classified as temperate

**Table S10:** Impact of increased crop diversity (M4) on SOC stock changes in absolute (ton ha^-1^ yr^-1^, ton ha^-1^) and relative terms (% yr^-1^, %) based on data from meta-studies. Management interventions are classified into crop rotation without cover crops (C), crop rotation + cover crops (CC) and perennial crop rotation (CCP). Climate zones are classified into temperate (temp), subtropical (subtr), tropical (trop)

| **Intervention** | **treatment - control** | **Climate zone** | **Ref no** | **∆ SOC over 20 years**  **in ton ha^-1^ yr^-1^ ± se** | **∆ SOC**  **in ton ha^-1^ yr^-1^ ± se** | **∆ SOC**  **in ton ha^-1^ ± se** | **∆ SOC**  **in % yr^-1^** | **∆ SOC**  **in % ± se** | **Average study duration in years [range]** | **Number of paired comparisons** | **Average sampling depth in m [range]** | **original effect size unit** | **Regional coverage** |
| --- | --- | --- | --- | --- | --- | --- | --- | --- | --- | --- | --- | --- | --- |
| C | Crop rotation - monoculture | temp | 12b | **-** | - | - | 0.20 | 3.6 | 18 | 251 | 0.25 | concentration | Global^3^ |
| CC | Crop rotation + cover crops - monoculture | temp | 12a | **-** | - | - | 0.47 | 8.5 | 18 | 81 | 0.25 | Concentration | Global^3^ |
|  | Crop rotation + cover crops - monoculture | temp | 16a | **-** | - | - | 0.50±0.25* | 10±4.96 | x | 13 | x | stock | Europe |
|  | Crop rotation + cover crops - monoculture | temp | 18b | 0.26±0.16** | 0.2±0.12 | 2.93±1.18 | - | - | 26 | 85 | 0.21 | stock | Global^3^ |
|  | Crop rotation + cover crops - monoculture (grain) | temp | 7a | 0.15** | 0.21* | 2.9 | 0.45* | 6.3 | 14 | 49 | [0-0.2] | stock | Global^3^ |
|  | Crop rotation + cover crops - no cover crops | subtr | 1b | 0.15±0.06** | 0.27±0.11 | 2.97±1.21* | 0.91±0.44* | 10±4.85 | 11 | 11 | 0.22 | stock | Mediterranean |
|  | Crop rotation + cover crops-no cover crops | trop | 6a | 0.17±0.03** | 0.69±0.10 | 3.45± 0.51* | - | 8.2±2.76 | 5^1^ | 114 | [0-0.3]^2^ | stock | Tropical |
|  |  | temp | 6b | 0.13±0.01** | 0.5±0.03 | 2.5 ± 0.15* | - | 16.6±1.07 | 5^1^ | 761 | [0-0.3]^2^ | stock | temperate |
|  | Crop rotation + cover crops - no cover crops | temp | 15a | 0.19±0.05** | 0.32±0.08 | 3.84±0.96* | - | - | 12 | 139 | 0.22 | stock | Global^3^ |
| CCP | Perennial crop rotation - monoculture (grain) | temp | 7b | 0.29** | 0.41* | 5.7 | 0.89* | 12.5 | 14 | 41 | [0-0.2] | stock | Global^3^ |

*Mean changes estimated based on average study durations provided by meta-study. If no average duration was provided, a time horizon of 20 years was assumed

**Normalized annual SOC stock changes over 20 years

^X^ Categories as defined by the studies: Study duration: <5; 5–10; 11–20; >20; Sampling depth: 0–10; 11–30; >30

^1^Most experiments reported after less than 5 years

^2^Sampling depth include surface (<30cm) and subsurface (>30cm), but most samples refer to <30cm

^3^Most studies conducted in north America and Europe, thus classified as temperate

**Table S11:** Impact of crop residue incorporation (M5) on SOC stock changes in absolute (ton ha^-1^ yr^-1^, ton ha^-1^) and relative terms (% yr^-1^, %) based on data from meta-studies. Management interventions are classified into crop residue incorporation vs. removal (CRES). Climate zones are classified into temperate (temp), subtropical (subtr), tropical (trop)

| **Intervention** | **treatment - control** | **Climate zone** | **Ref no** | **∆ SOC over 20 years**  **in ton ha^-1^ yr^-1^ ± se** | **∆ SOC**  **in ton ha^-1^ yr^-1^ ± se** | **∆ SOC**  **in ton ha^-1^ ± se** | **∆ SOC**  **in % yr^-1^** | **∆ SOC**  **in % ± se** | **Average study duration in years [range]** | **Number of paired comparisons** | **Average sampling depth in m [range]** | **original effect size unit** | **Regional coverage** |
| --- | --- | --- | --- | --- | --- | --- | --- | --- | --- | --- | --- | --- | --- |
| CRES | Corn stover retention - removal | temp | 19a | 0.21± 0.02** | 0.41±0.04 | 4.1±0.38* | - | - | 10^2^ | 110 | 0.3^3^ | stock | Global^1^ |
| CRES | Crop residue retention - removal | temp | 9a | - | - | - | 0.35±0.07* | 7±1.39 | x | 84 | x | Concentration | Europe |

*Mean changes estimated based on average study durations provided by meta-study. If no average duration was provided, a time horizon of 20 years was assumed

**Normalized annual SOC stock changes over 20 years

^x^ Categories as defined by the studies: Study duration: <5; 5–10; 11–20; >20; Sampling depth: 0–10; 11–30; >30

^1^Most data from global temperate regions (e.g. north America and or Europe), therefore classified as temperate

^2^Study duration approximated from Figure 2a where, 0-5 yrs (50 %) and 5-20 yrs (50%).

^3^Sampling depth mostly refers to 0-30cm. Part of data included refer to >30

**Supplementary figures**

**
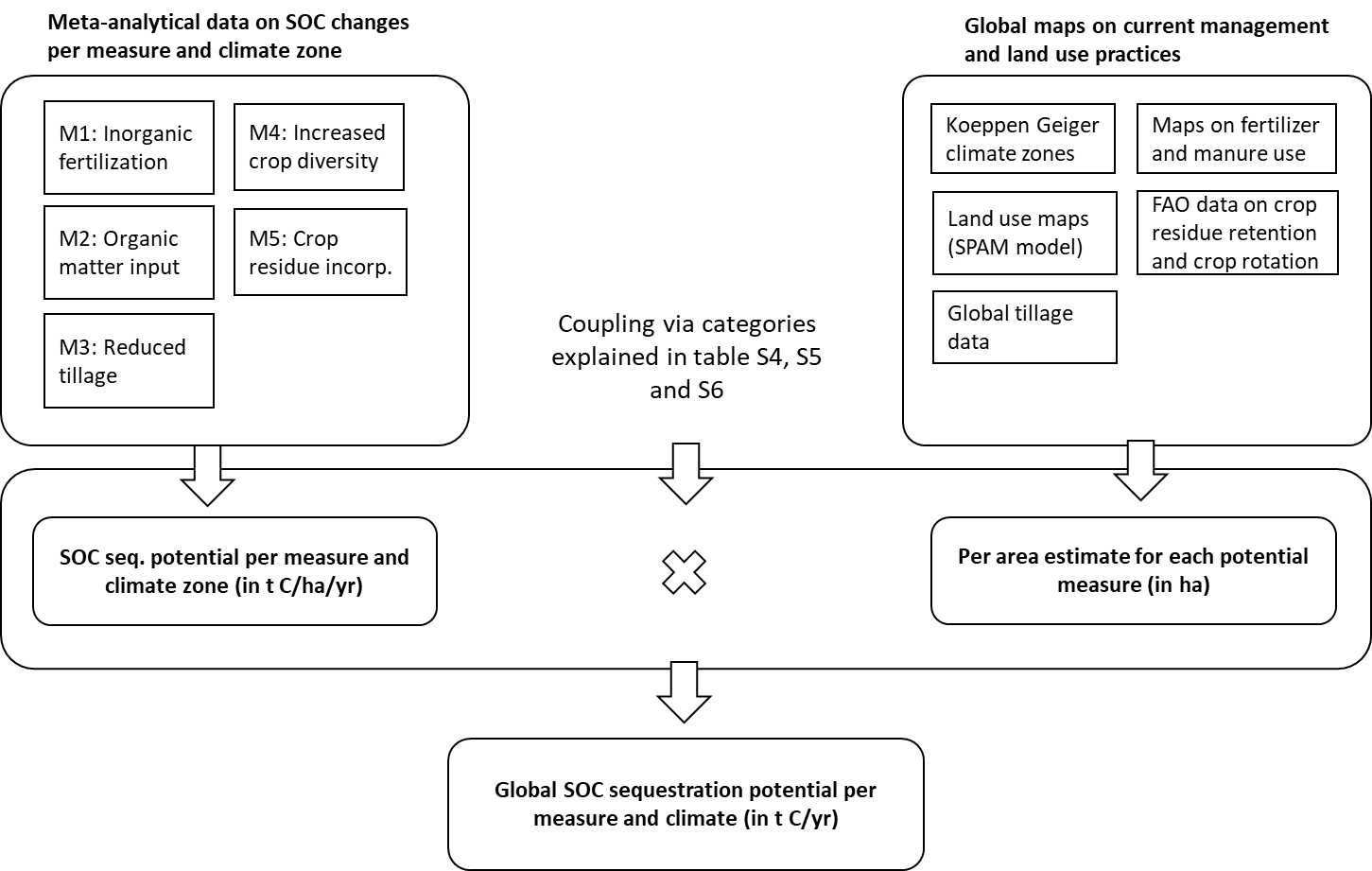
Figure S1:** Flow chart depicting the upscaling process to link meta-analytical data on SOC changes per management and climate to global maps on management and land use practices to derive global SOC sequestration potentials per management and climate.


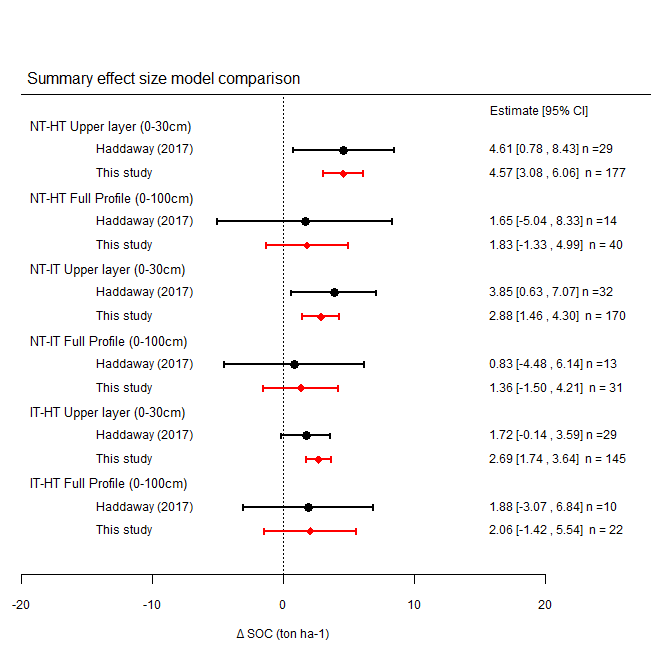


**Figure S2**: Impacts of converting data on SOC concentration changes to stock changes (t C ha^-1^) from tillage experiments from Haddaway et al.(2017). The original SOC stock change estimates (shown in black) are compared with the converted SOC stock changes (red) when combining original field study data on SOC concentration with spatially-explicit and soil-layer dependent bulk density estimates from SoilGrids (Hengl et al., 2014). The stock changes are depicted for different tillage interventions No-Till relative to High Intensity-Till (NT-HT), No-Till relative to Inversion-Till (NT-IT) and Inversion-Till relative to High Intensity-Till (IT-HT) and depth layers. For the global upscaling approach, only data for the 0-30cm soil depth were used.

**Supplementary References**

Aguilera, E., Lassaletta, L., Gattinger, A., & Gimeno, B. S. (2013). Managing soil carbon for climate change mitigation and adaptation in Mediterranean cropping systems: A meta-analysis. *Agriculture, Ecosystems and Environment*, *168*, 25–36. https://doi.org/10.1016/j.agee.2013.02.003

Angers, D. A., & Eriksen-Hamel, N. S. (2008). Full-Inversion Tillage and Organic Carbon Distribution in Soil Profiles: A Meta-Analysis. *Soil Science Society of America Journal*, *72*(5), 1370. https://doi.org/10.2136/sssaj2007.0342

Cooper, J., Baranski, M., Stewart, G., Nobel-de Lange, M., Bàrberi, P., Fließbach, A., Peigné, J., Berner, A., Brock, C., Casagrande, M., Crowley, O., David, C., De Vliegher, A., Döring, T. F., Dupont, A., Entz, M., Grosse, M., Haase, T., Halde, C., … Mäder, P. (2016). Shallow non-inversion tillage in organic farming maintains crop yields and increases soil C stocks: a meta-analysis. In *Agronomy for Sustainable Development* (Vol. 36, Issue 1). Agronomy for Sustainable Development. https://doi.org/10.1007/s13593-016-0354-1

Haddaway, N. R., Hedlund, K., Jackson, L. E., Kätterer, T., Lugato, E., Thomsen, I. K., Jørgensen, H. B., & Isberg, P. E. (2017). How does tillage intensity affect soil organic carbon? A systematic review. In *Environmental Evidence* (Vol. 6, Issue 1). https://doi.org/10.1186/s13750-017-0108-9

Han, P., Zhang, W., Wang, G., Sun, W., & Huang, Y. (2016). Changes in soil organic carbon in croplands subjected to fertilizer management: a global meta-analysis. *Scientific Reports*, *6*(February), 27199. https://doi.org/10.1038/srep27199

Hengl, T., Jesus, J. M. De, Macmillan, R. A., Batjes, N. H., Heuvelink, G. B. M., Ribeiro, E., Samuel-rosa, A., Kempen, B., Leenaars, J. G. B., Walsh, M. G., & Gonzalez, M. R. (2014). *SoilGrids1km — Global Soil Information Based on Automated Mapping*. *9*(8). https://doi.org/10.1371/journal.pone.0105992

Jian, J., Du, X., Reiter, M. S., & Stewart, R. D. (2020). A meta-analysis of global cropland soil carbon changes due to cover cropping. *Soil Biology and Biochemistry*, *143*. https://doi.org/10.1016/j.soilbio.2020.107735

King, A. E., & Blesh, J. (2018). Crop rotations for increased soil carbon: perenniality as a guiding principle. *Ecological Applications*, *28*(1), 249–261. https://doi.org/10.1002/eap.1648

Ladha, J. K., Reddy, C. K., Padre, A. T., & van Kessel, C. (2011). Role of Nitrogen Fertilization in Sustaining Organic Matter in Cultivated Soils. *Journal of Environment Quality*, *40*(6), 1756. https://doi.org/10.2134/jeq2011.0064

Lehtinen, T., Schlatter, N., Baumgarten, A., Bechini, L., Krüger, J., Grignani, C., Zavattaro, L., Costamagna, C., & Spiegel, H. (2014). Effect of crop residue incorporation on soil organic carbon and greenhouse gas emissions in European agricultural soils. *Soil Use and Management*, *30*(4), 524–538. https://doi.org/10.1111/sum.12151

Lu, M., Zhou, X., Luo, Y., Yang, Y., Fang, C., Chen, J., & Li, B. (2011). Minor stimulation of soil carbon storage by nitrogen addition: A meta-analysis. *Agriculture, Ecosystems and Environment*, *140*(1–2), 234–244. https://doi.org/10.1016/j.agee.2010.12.010

Maillard, É., & Angers, D. A. (2014). Animal manure application and soil organic carbon stocks: A meta-analysis. *Global Change Biology*, *20*(2), 666–679. https://doi.org/10.1111/gcb.12438

McDaniel, M. D. M., Tiemann, L. K. L., & Grandy, A. S. (2014). Does agricultural crop diversity enhance soil microbial biomass and organic matter dynamics? a meta-analysis. *Ecological Applications*, *24*(3), 560–570. https://doi.org/10.1890/13-0616.1

Meurer, K. H. E., Haddaway, N. R., Bolinder, M. A., & Kätterer, T. (2018). Tillage intensity affects total SOC stocks in boreo-temperate regions only in the topsoil—A systematic review using an ESM approach. *Earth-Science Reviews*, *177*, 613–622. https://doi.org/10.1016/J.EARSCIREV.2017.12.015

Ogle, S. M. S. M., Breidt, F. J. J. F. J., & Paustian, K. (2005). Agricultural management impacts on soil organic carbon storage under moist and dry climatic conditions of temperate and tropical regions. *Biogeochemistry*, *72*(1), 87–121. https://doi.org/10.1007/s10533-004-0360-2

Poeplau, C., & Don, A. (2015). Carbon sequestration in agricultural soils via cultivation of cover crops - A meta-analysis. *Agriculture, Ecosystems and Environment*, *200*, 33–41. https://doi.org/10.1016/j.agee.2014.10.024

Spiegel, H., Schlatter, N., Haslmayr, H.-P., Lehtinen, T., & Baumgarten, A. (2014). *Compatibility of Agricultural Management Practices and Types of Farming in the EU to enhance Climate Change Mitigation and Soil Health Policy bundles framing agricultural soil protection in EU and selected member states*. *January 2012*.

Virto, I., Barré, P., Burlot, A., & Chenu, C. (2012). Carbon input differences as the main factor explaining the variability in soil organic C storage in no-tilled compared to inversion tilled agrosystems. *Biogeochemistry*, *108*(1–3), 17–26. https://doi.org/10.1007/s10533-011-9600-4

West, T. O., & Post, W. M. (2002). Soil organic carbon sequestration rates by tillage and crop rotation: A global data analysis. *Soil Science Society of America Journal*, *66*(6), 1930–1946. https://doi.org/10.3334/CDIAC/tcm

Xu, H., Sieverding, H., Kwon, H., Clay, D., Stewart, C., Johnson, J. M. F., Qin, Z., Karlen, D. L., & Wang, M. (2019). A global meta-analysis of soil organic carbon response to corn stover removal. *GCB Bioenergy*, *11*(10), 1215–1233. https://doi.org/10.1111/gcbb.12631

Zavattaro, L., Bechini, L., Grignani, C., van Evert, F. K., Mallast, J., Spiegel, H., Sandén, T., Pecio, A., Giráldez Cervera, J. V., Guzmán, G., Vanderlinden, K., D’Hose, T., Ruysschaert, G., ten Berge, H. F. M., D’Hose, T., Ruysschaert, G., & ten Berge, H. F. M. (2017). Agronomic effects of bovine manure: A review of long-term European field experiments. *European Journal of Agronomy*, *90*, 127–138. https://doi.org/10.1016/j.eja.2017.07.010
